# Supplementary material for: Six New Phenylpropanoid Derivatives from Chemically Converted Extract of Alpinia galanga (L.) and Their Antiparasitic Activities
Source: Molecules. 2021 Mar 21;26(6):1756. doi: 10.3390/molecules26061756 (PMC8004034; doi:10.3390/molecules26061756)
Supplement: Supplementary file 1 [file molecules-26-01756-s001.pdf]

Supplementary Material

Six New Phenylpropanoid Derivatives from Chemically Converted Extract of *Alpinia galanga* (L.) and their anti-parasitic activities

Melanny Ika Sulistyowaty[1,2], Nguyen Hoang Uyen[1], Keisuke Suganuma[3], Ben-Yeddy Abel Chitama[4], Kazuhide Yahata[4], Osamu Kaneko[4], Sachiko Sugimoto[1], Yoshi Yamano[1], Susumu Kawakami[5], Hideaki Otsuka[5] and Katsuyoshi Matsunami[1,*]

1 Graduate School of Biomedical and Health Sciences, Hiroshima University, Hiroshima, Japan; melanny-i-s@ff.unair.ac.id (M.I.S.); kimdonguyen452@yahoo.com (N.H.U); ssugimot@hiroshima-u.ac.jp (S.S.); yamano@hiroshima-u.ac.jp (Y.Y.); matunami@hiroshima-u.ac.jp (K.M.)

2 Faculty of Pharmacy, Universitas Airlangga, Surabaya, Indonesia; melanny-i-s@ff.unair.ac.id (M.I.S.)

3 National Research Center for Protozoan Diseases, Obihiro University of Agriculture and Veterinary Medicine, Inada, Obihiro, Hokkaido, 080-8555, Japan; k.suganuma@obihiro.ac.jp (K.S.)

4 Department of Protozoology, Institute of Tropical Medicine (NEKKEN), Nagasaki University, 1-12-4 Sakamoto, Nagasaki 852-8523, Japan; okaneko@nagasaki-u.ac.jp (O.K.); kyahata@nagasaki-u.ac.jp (K.Y.); benchitama@gmail.com (BY.A.C.)

5 Department of Natural Products Chemistry, Faculty of Pharmacy, Yasuda Women's University, 6-13-1 Yasuhigashi, Asaminami-ku, Hiroshima 731-0153, Japan; kawakami@yasuda-u.ac.jp (S.K.); otsuka-h@yasuda-u.ac.jp (H.O.)

* Correspondence: matunami@hiroshima-u.ac.jp; Tel.: (+81-82-257-5335)(K.M.)

Contents:

**Figure S1**	**HPLC chromatogram for the isolation of 1, 7 and 8**
**Figure S2**	**HPLC chromatogram for the isolation of 1, 2, 6, 9 and 10**
**Figure S3**	**HPLC chromatogram for the isolation of 3, 4, 5 and 7**
**Figure S4**	**HR-ESI-MS of 1**
**Figure S5**	**^{1}H NMR spectrum of 1**
**Figure S6**	**^{13}C NMR spectrum of 1**
**Figure S7**	**DEPT 135 spectrum of 1**
**Figure S8**	**COSY spectrum of 1**
**Figure S9**	**HSQC spectrum of 1**
**Figure S10**	**HMBC spectrum of 1**
**Figure S11**	**IR spectrum of 1**
**Figure S12**	**HR-ESI-MS of 2**
**Figure S13**	**^{1}H NMR spectrum of 2**
**Figure S14**	**^{13}C NMR spectrum of 2**
**Figure S15**	**DEPT 135 spectrum of 2**
**Figure S16**	**COSY spectrum of 2**
**Figure S17**	**HSQC spectrum of 2**
**Figure S18**	**HMBC spectrum of 2**
**Figure S19**	**IR spectrum of 2**
**Figure S20**	**HR-ESI-MS of 3**
**Figure S21**	**^{1}H NMR spectrum of 3**
**Figure S22**	**^{13}C NMR spectrum of 3**
**Figure S23**	**DEPT 135 spectrum of 3**
**Figure S24**	**COSY spectrum of 3**
**Figure S25**	**HSQC spectrum of 3**
**Figure S26**	**HMBC spectrum of 3**
**Figure S27**	**IR spectrum of 3**
**Figure S28**	**HR-ESI-MS of 4**
**Figure S29**	**^{1}H NMR spectrum of 4**
**Figure S30**	**^{13}C NMR spectrum of 4**
**Figure S31**	**DEPT 135 spectrum of 4**
**Figure S32**	**COSY spectrum of 4**
**Figure S33**	**HSQC spectrum of 4**
**Figure S34**	**HMBC spectrum of 4**
**Figure S35**	**IR spectrum of 4**
**Figure S36**	**HR-ESI-MS of 5**
**Figure S37**	**^{1}H NMR spectrum of 5**
**Figure S38**	**^{13}C NMR spectrum of 5**
**Figure S39**	**DEPT 135 spectrum of 5**
**Figure S40**	**COSY spectrum of 5**
**Figure S41**	**HSQC spectrum of 5**

**Figure S42**	**HMBC spectrum of 5**
**Figure S43**	**IR spectrum of 5**
**Figure S44**	**HR-ESI-MS of 6**
**Figure S45**	**^{1}H NMR spectrum of 6**
**Figure S46**	**^{13}C NMR spectrum of 6**
**Figure S47**	**DEPT 135 spectrum of 6**
**Figure S48**	**COSY spectrum of 6**
**Figure S49**	**HSQC spectrum of 6**
**Figure S50**	**HMBC spectrum of 6**
**Figure S51**	**IR spectrum of 6**
**Figure S52**	**^{1}H NMR spectrum of 7**
**Figure S53**	**^{13}C NMR spectrum of 7**
**Figure S54**	**^{1}H NMR spectrum of 8**
**Figure S55**	**^{13}C NMR spectrum of 8**
**Figure S56**	**^{1}H NMR spectrum of 9**
**Figure S57**	**^{13}C NMR spectrum of 9**
**Figure S58**	**^{1}H NMR spectrum of 10**
**Figure S59**	**^{13}C NMR spectrum of 10**
**Figure S60**	**Structures of positive control for the bioassay**
**Table S1**	**COSY correlations of compounds 1-6**
**Table S2**	**HMBC correlations of compounds 1-6**

Figure S1 HPLC chromatogram for the isolation of 1, 7 and 8

20% aq. acetone

Peak 1 = Cpd **7** (t_R = 10.7 min)

Peak 2 = Cpd **8** (t_R = 36.5 min)

Peak 3 = Cpd **1** (t_R = 45.3 min)

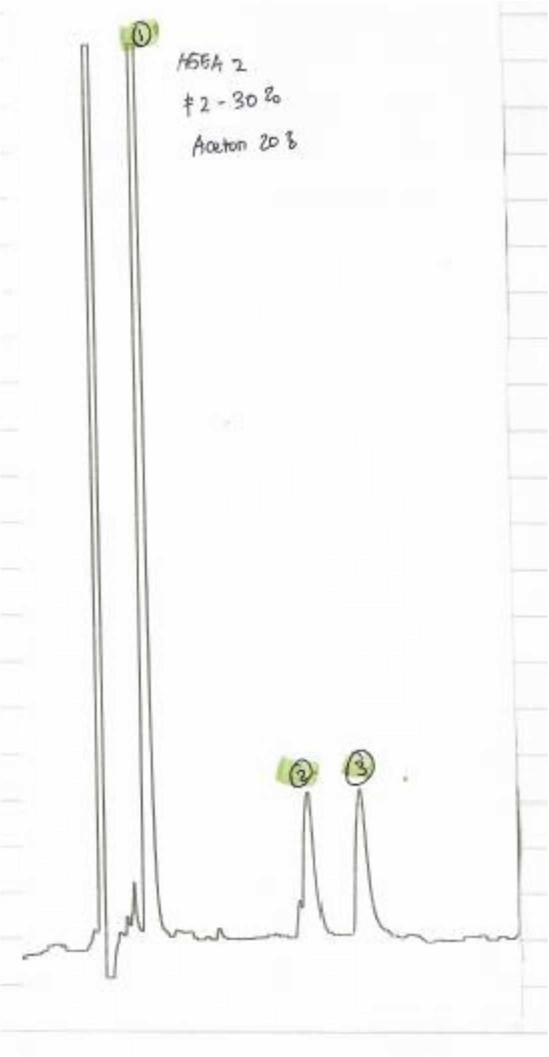

Figure S2 HPLC chromatogram for the isolation of 1, 2, 6, 9 and 10

30% aq. MeOH

Peak 1 = Cpd **1** (t_R = 18.7 min)

Peak 2 = Cpd **9** (t_R = 26.5 min)

Peak 3 = Cpd **2** (t_R = 30.2 min)

Peak 4 = Cpd **6** (t_R = 38.4 min)

Peak 5 = Cpd **10** (t_R = 46.3 min)

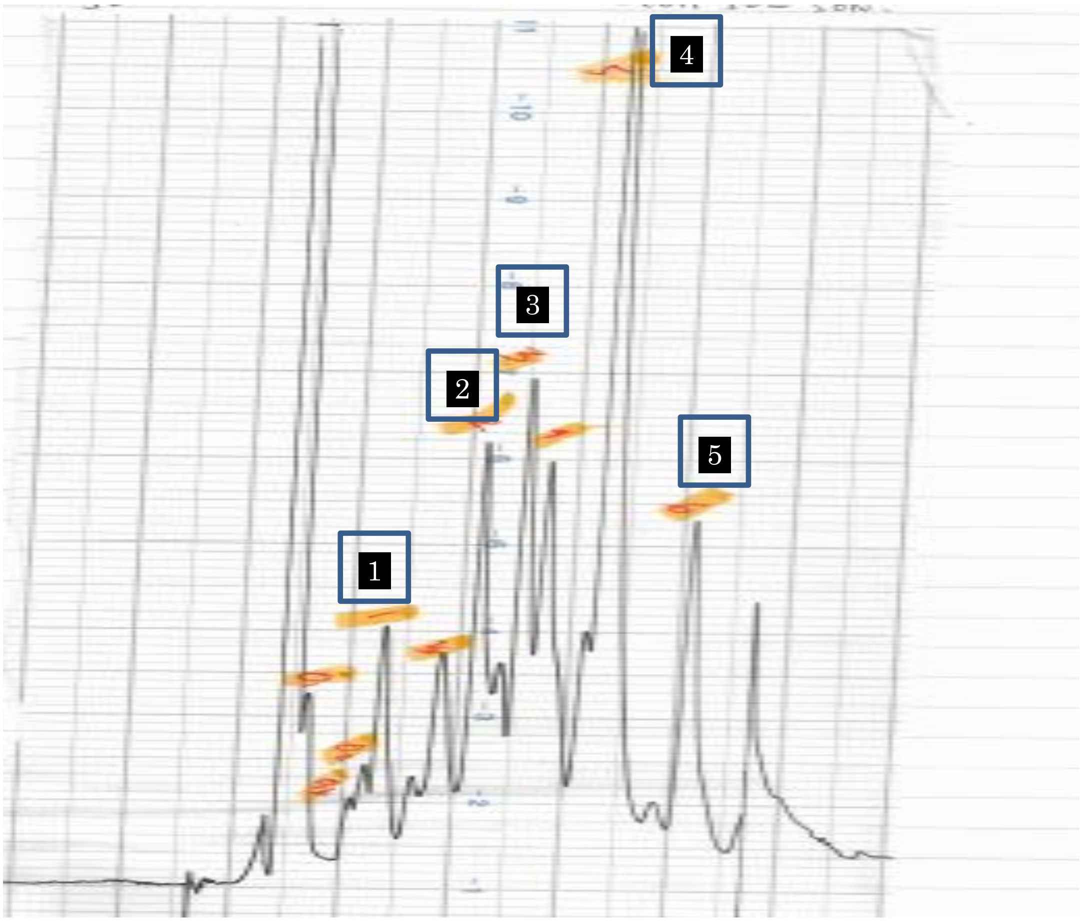

Figure S3 **HPLC chromatogram for the isolation of 3, 4, 5 and 7**

20% aq. acetone

Peak 1 = Cpd **3** (t_R = 9.5 min)

Peak 2 = Cpd **7** (t_R = 12.7 min)

Peak 3 = Cpd **4** (t_R = 16.3 min)

Peak 4 = Cpd **5** (t_R = 22.8 min)

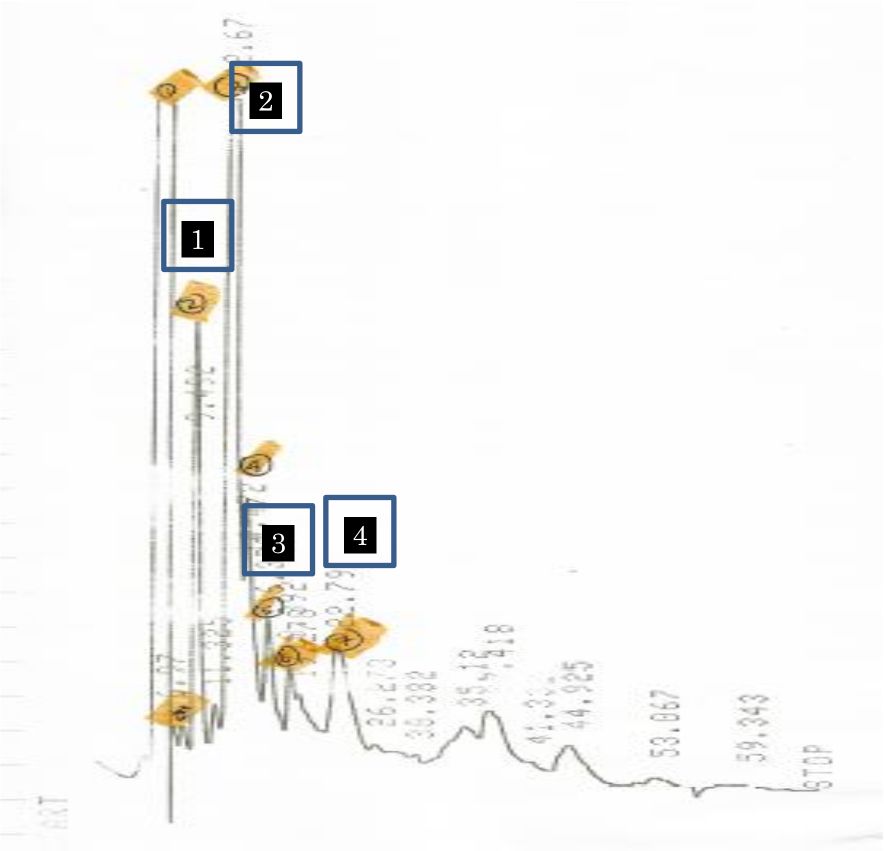

Figure S4 HR-ESI-MS of 1

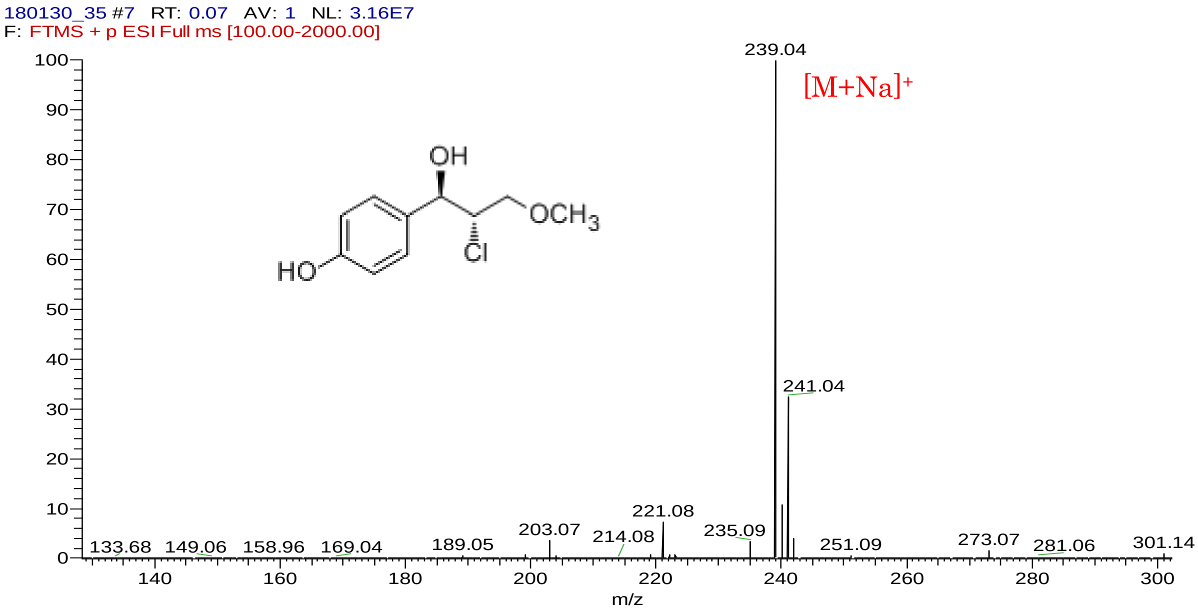

Elemental composition search on mass 239.04

m/z= 234.04-244.04

m/z	Theo. Mass	Delta (ppm)	RDB equiv.	Composition
239.0446	239.0445	0.36	3.5	$C_{10} H_{13} O_3 Cl Na$
[illegible]	[illegible]	[illegible]	[illegible]	[illegible]

Figure S5 ^{1}H NMR spectrum of 1

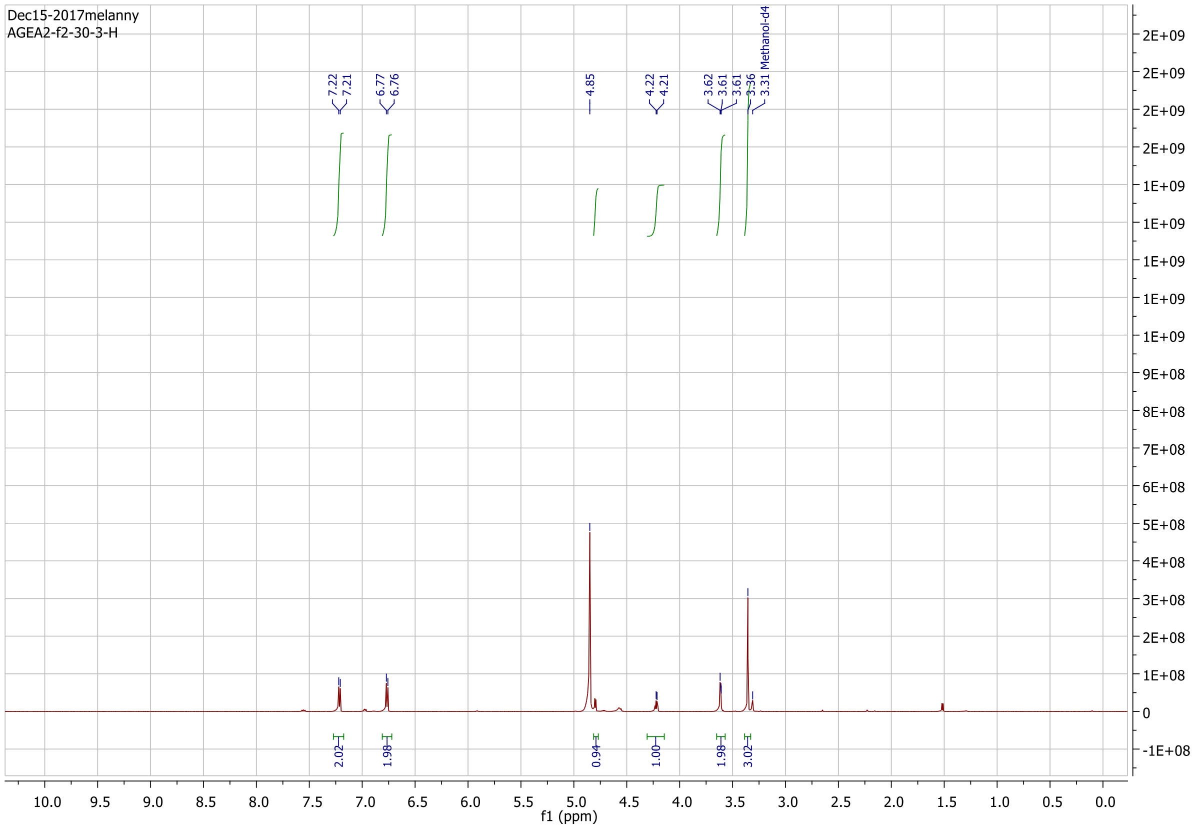

Figure S6 ^{13}C NMR spectrum of 1

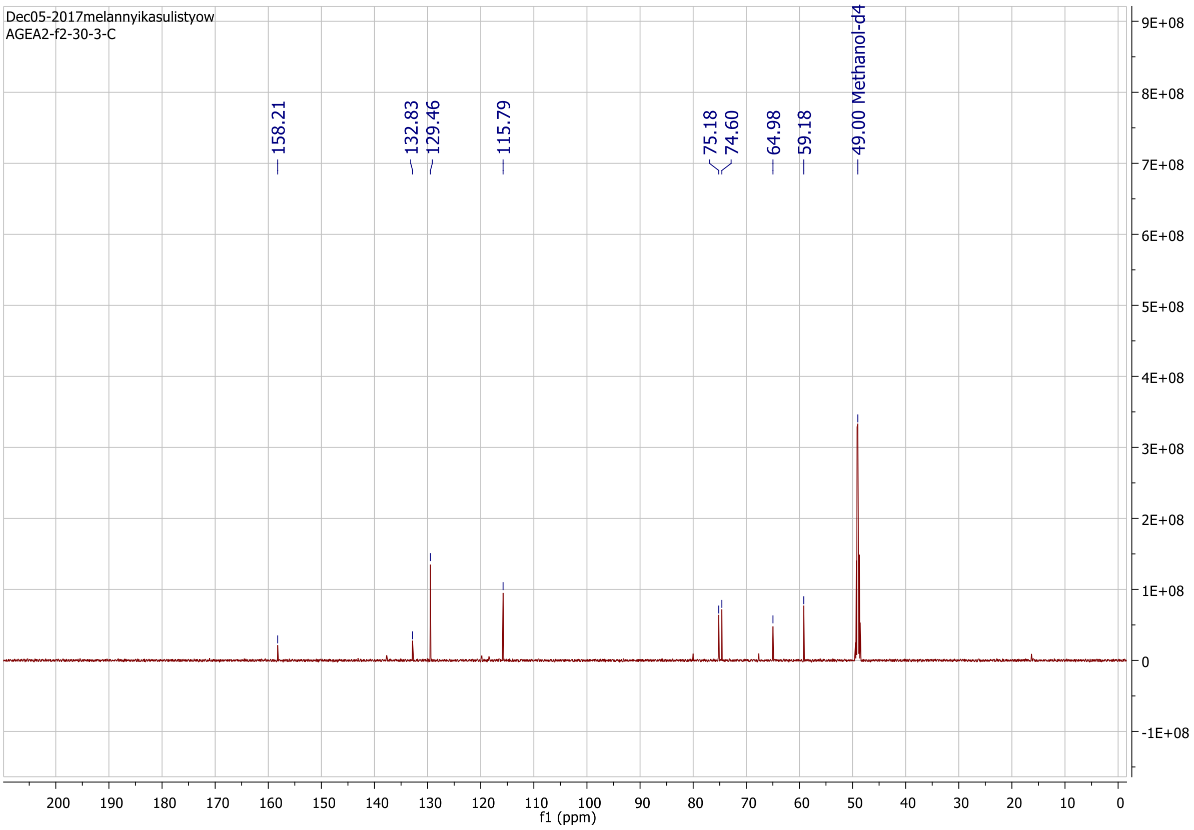

Figure S7 DEPT 135 spectrum of 1

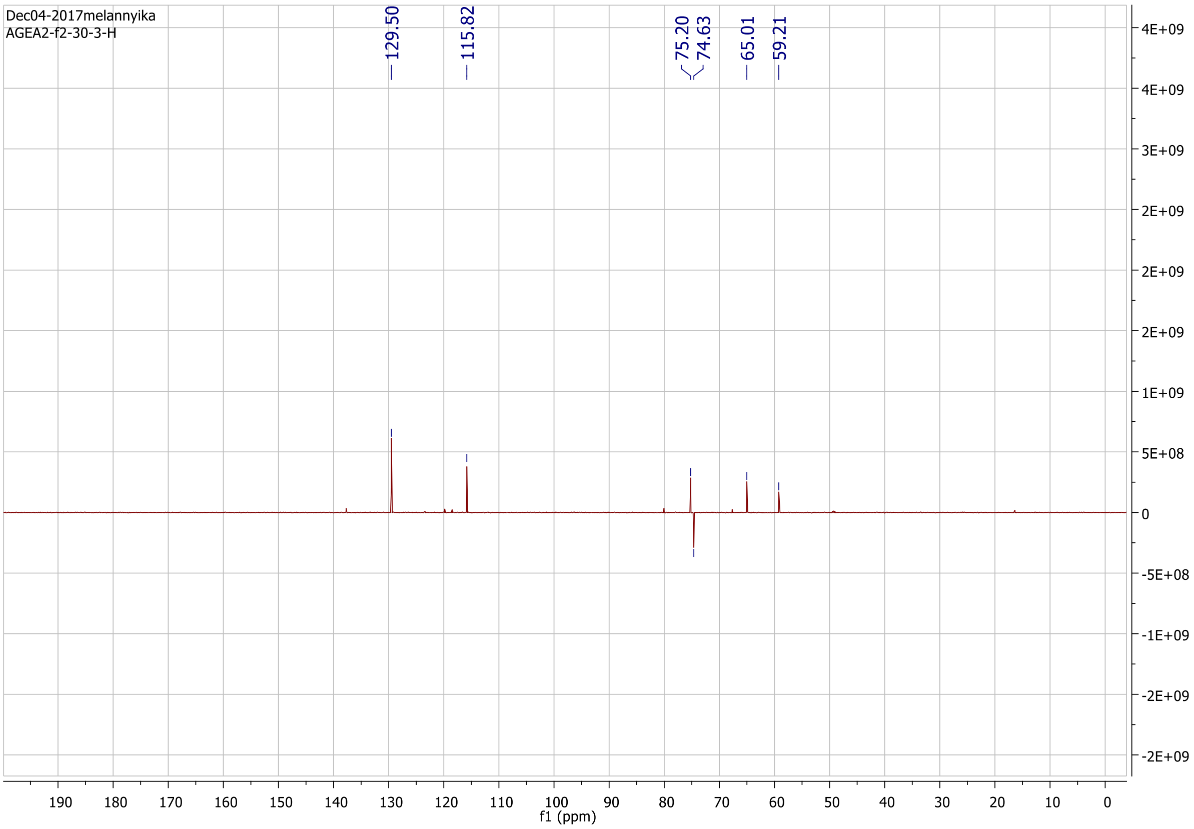

Figure S8 COSY spectrum of 1

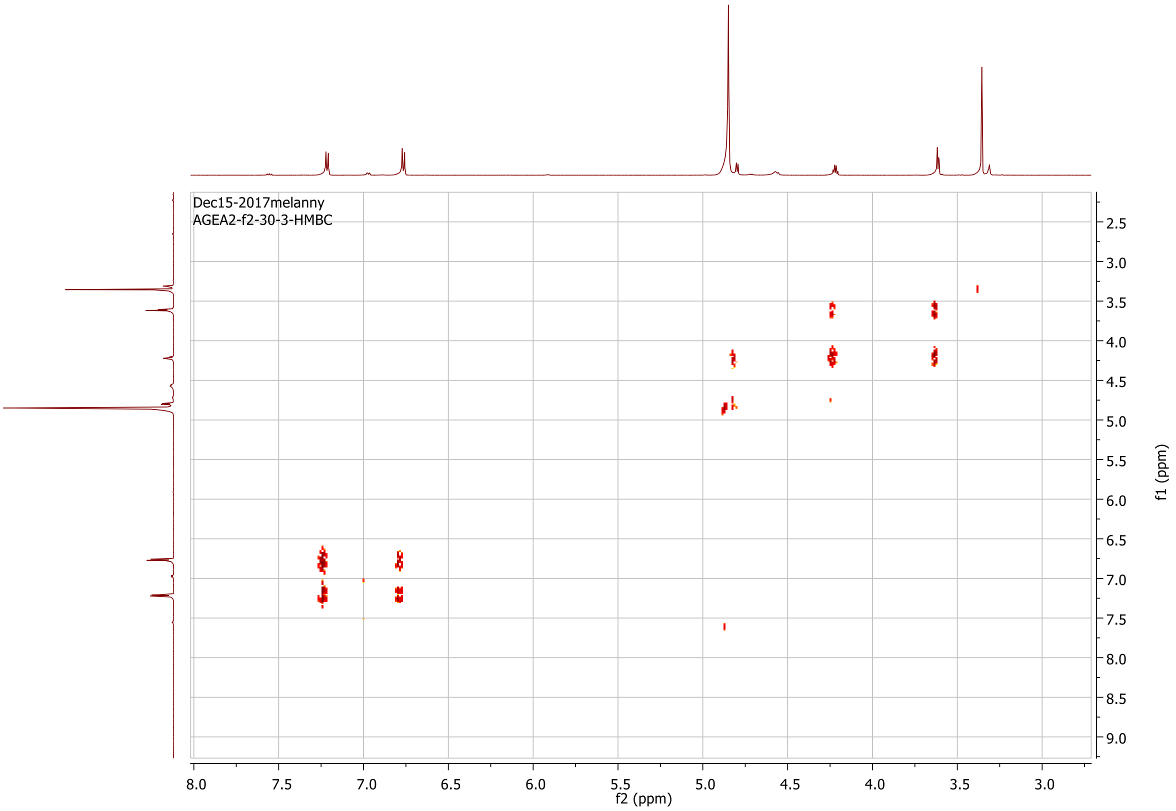

Figure S9 HSQC spectrum of 1

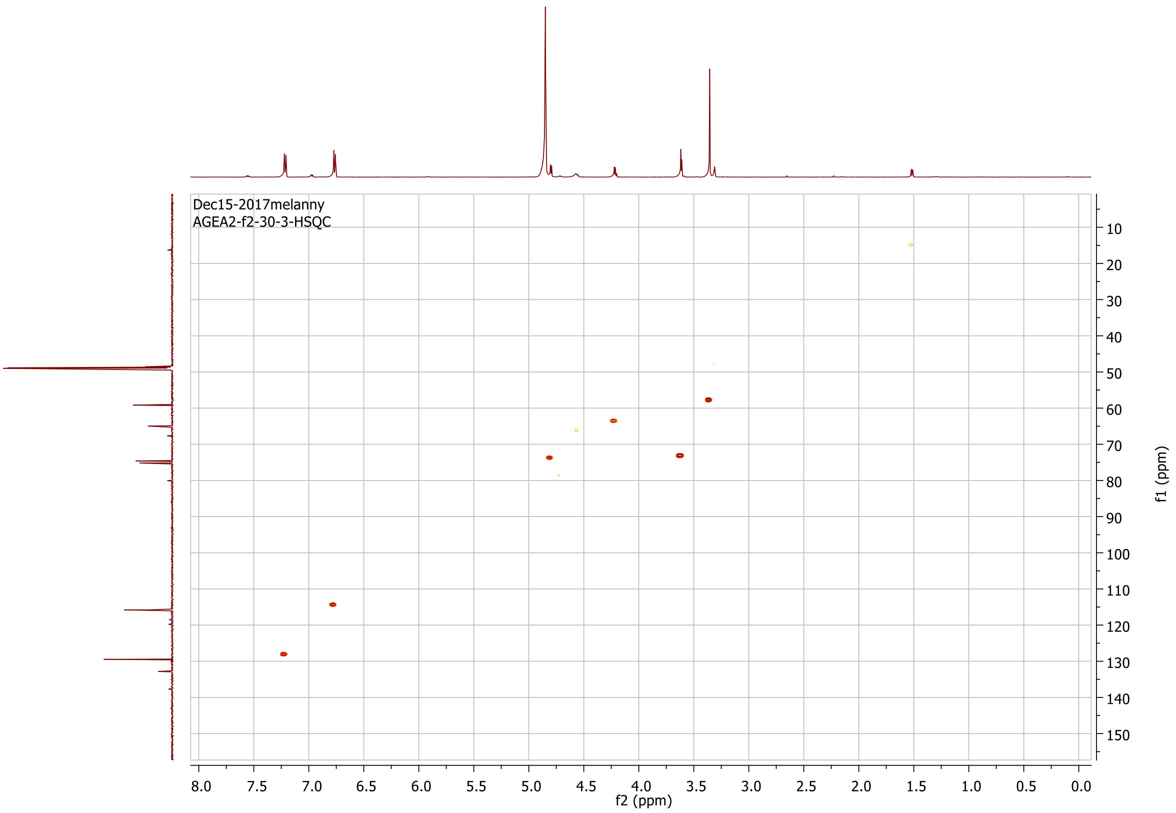

Figure S10 HMBC spectrum of 1

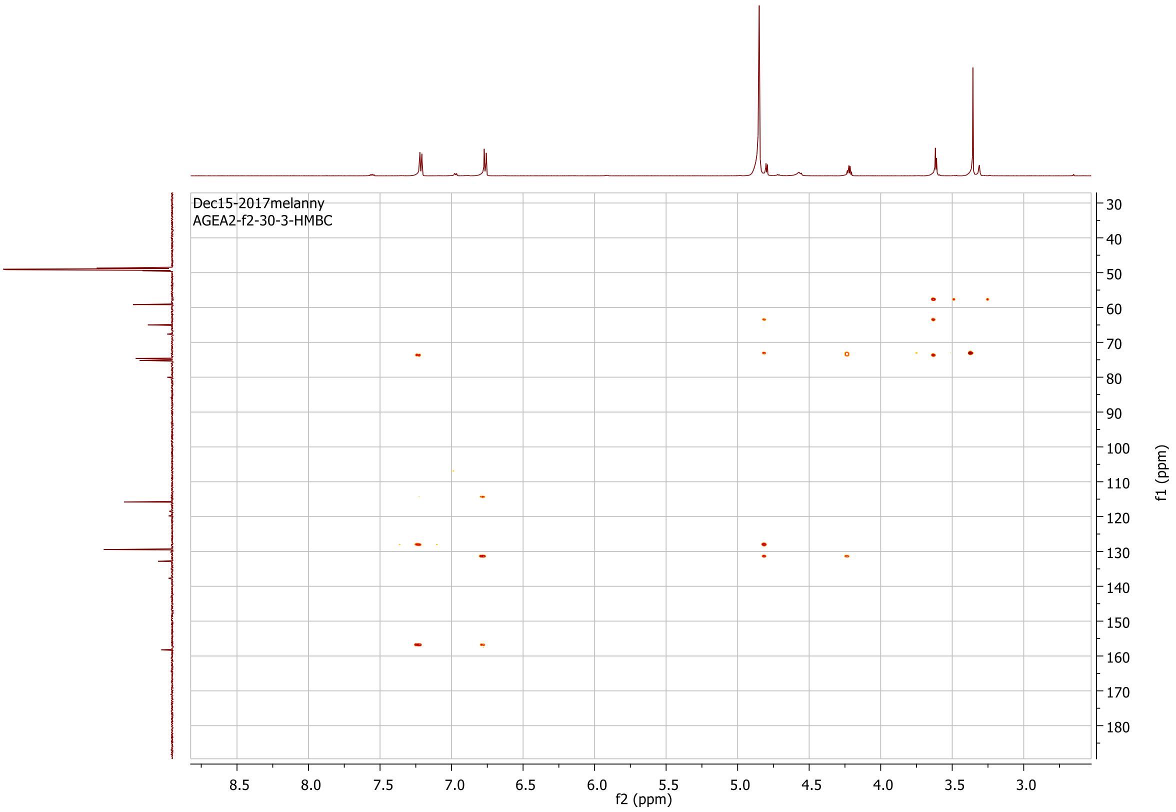

Figure S11 IR spectrum of 1

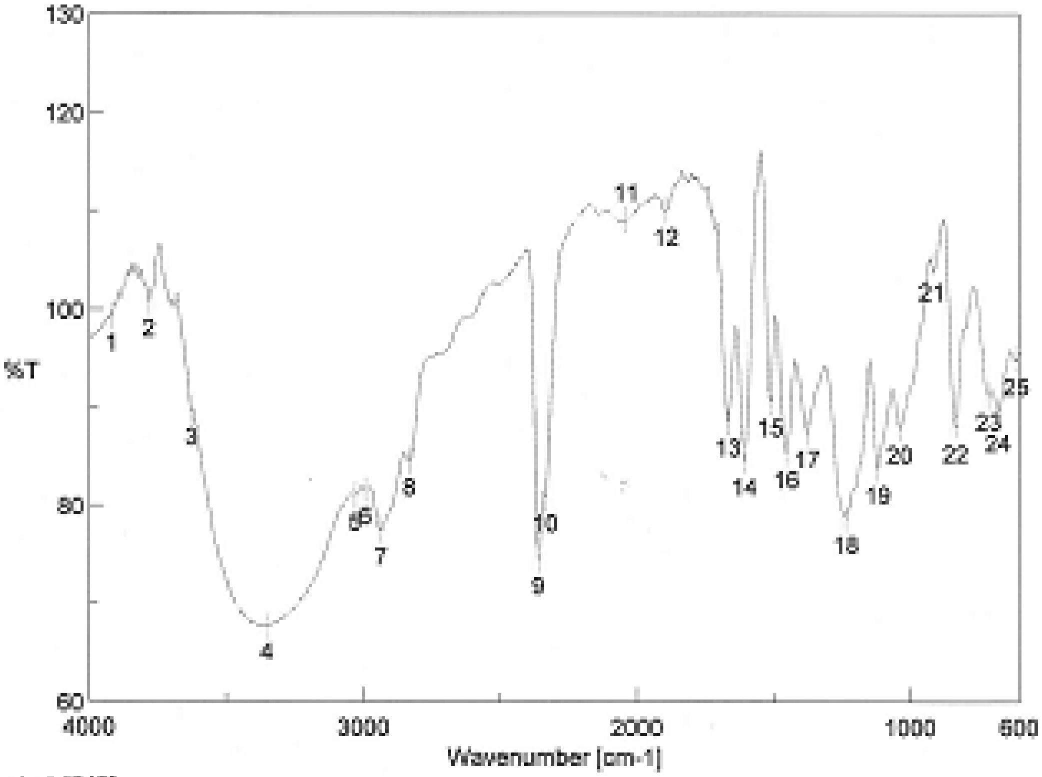

[コメント情報]
試料名
コメント
測定者
所属
会社

[データ情報]		[測定情報]	
作成日時	2018/08/20 13:05	機種名	FT/IR-4600typeA
		シリアル番号	D015461788
データタイプ	等間隔データ		
横軸	Wavenumber [cm-1]	測定日時	2018/08/20 12:59
縦軸	%T		
スタート	599.753 cm-1	光源	標準光源
エンド	7800.65 cm-1	検出器	TGS
データ間隔	0.964233 cm-1	積算回数	10
データ数	7469	分解	4 cm-1
		ゼロフィリング	On
		アポダイゼーション	Cosine
		ゲイン	Auto (2)
		アパーチャー	Auto (7.1 mm)
		スキャンスピード	Auto (2 mm/sec)
		フィルタ	Auto (30000 Hz)

[ピーク検出結果]

No.	位置	強度	No.	位置	強度
1	3918.64	99.2909	2	3761.72	100.787
3	3628.41	89.708	4	3350.71	67.8305
5	3030.59	81.0018	6	2992.02	81.6134
7	2935.13	77.4889	8	2831.96	84.5991
9	2360.44	74.5306	10	2335.37	80.9006
11	2040.32	109.045	12	1893.75	110.019
13	1668.12	88.6298	14	1607.38	84.6285

Figure S12 HR-ESI-MS of 2

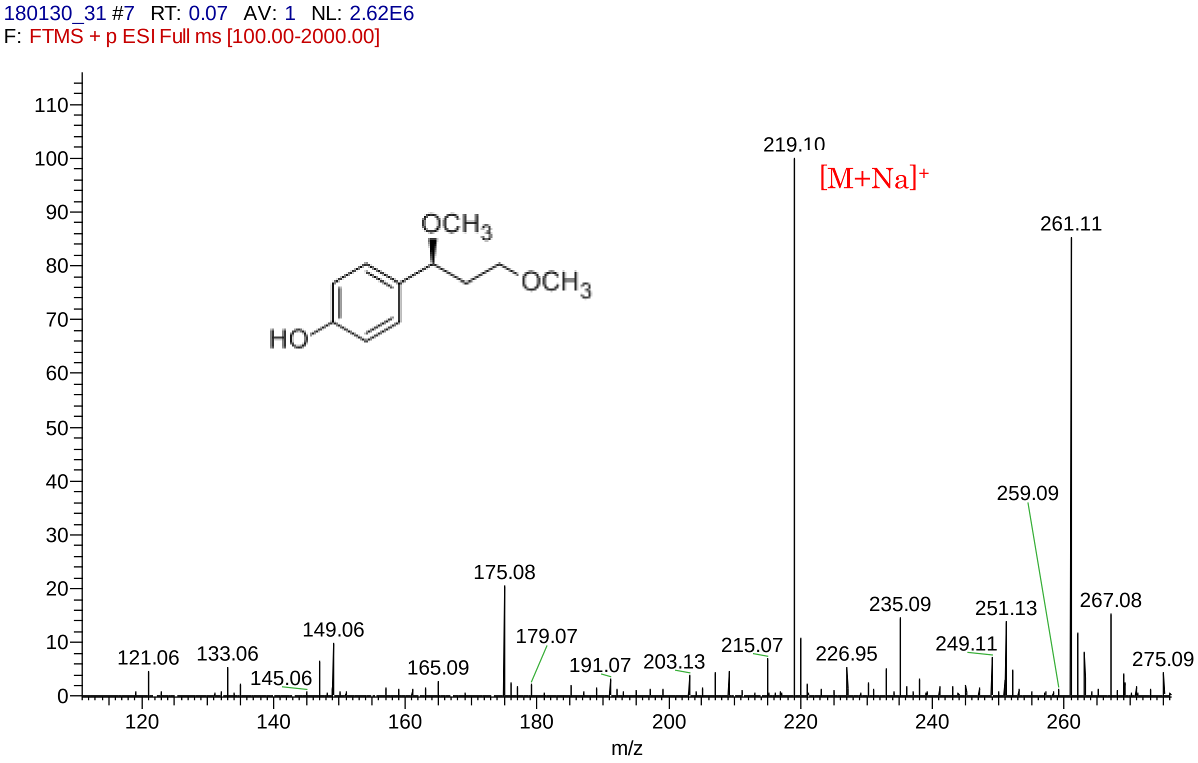

Elemental composition search on mass 219.10

m/z= 214.10-224.10

m/z	Theo. Mass	Delta (ppm)	RDB equiv.	Composition
219.0991	219.0992	-0.21	3.5	$C_{11}H_{16}O_3Na$

Figure S13 ^{1}H NMR spectrum of 2

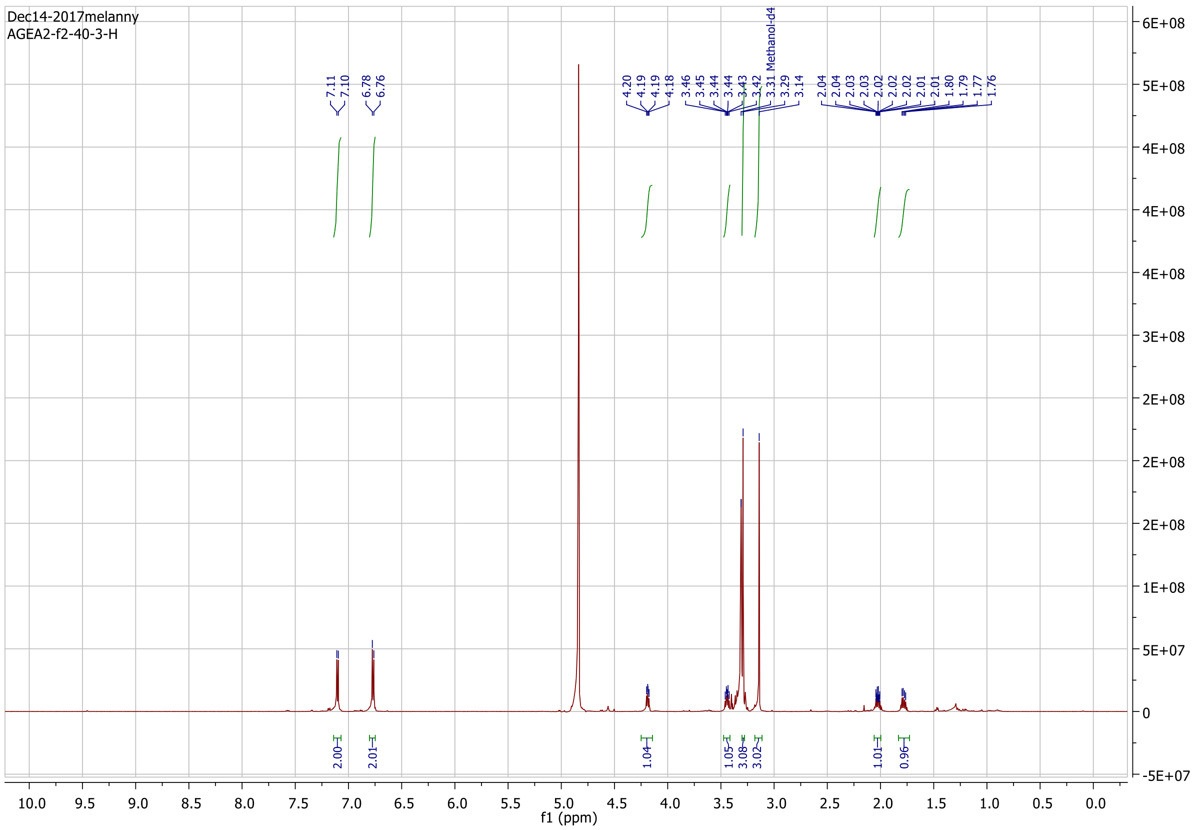

Figure S14 ^{13}C NMR spectrum of **2**

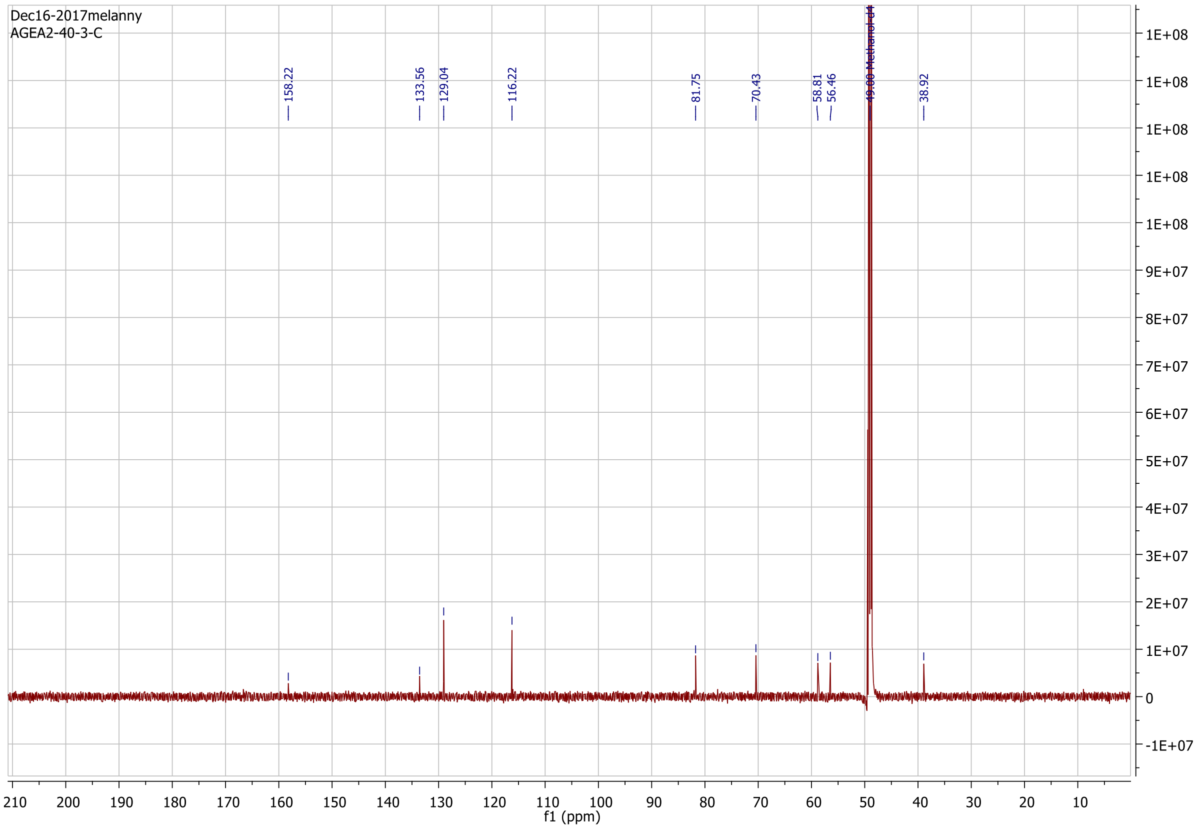

Figure S15 DEPT 135 spectrum of 2

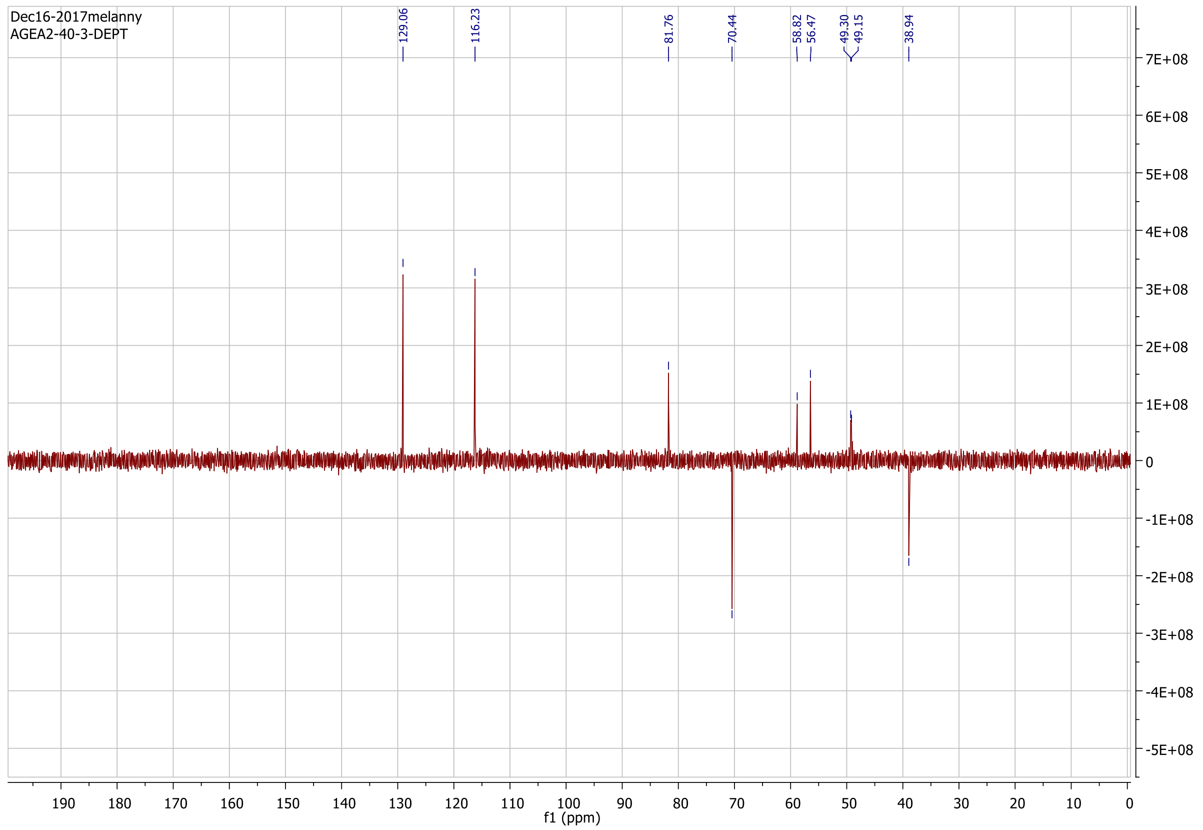

Figure S16 COSY spectrum of 2

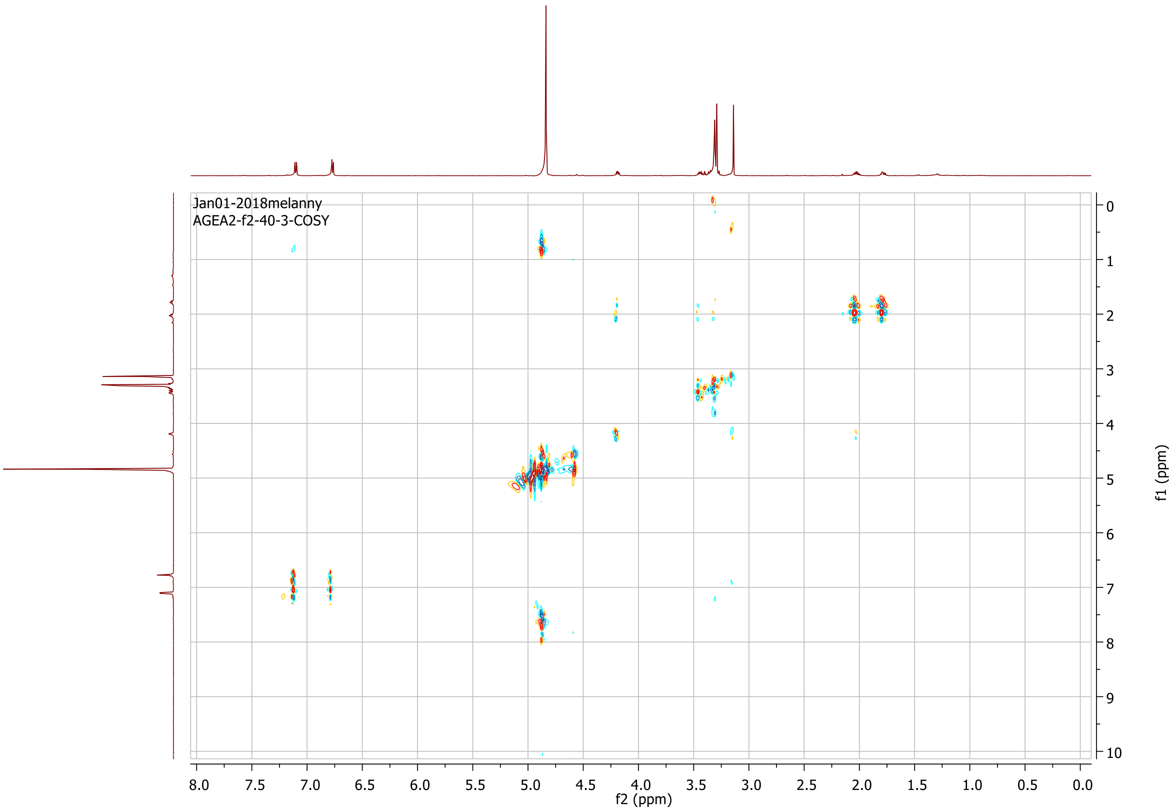

Figure S17 HSQC spectrum of 2

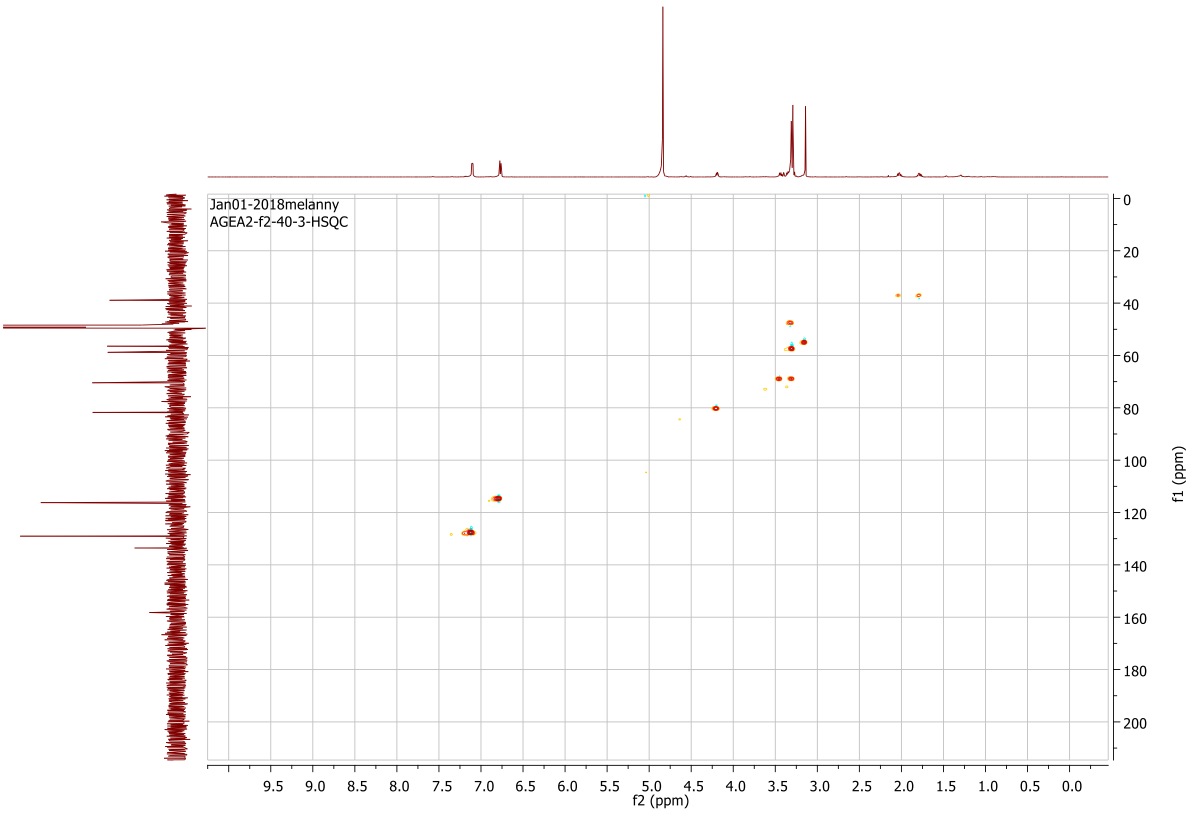

Figure S18 HMBC spectrum of 2

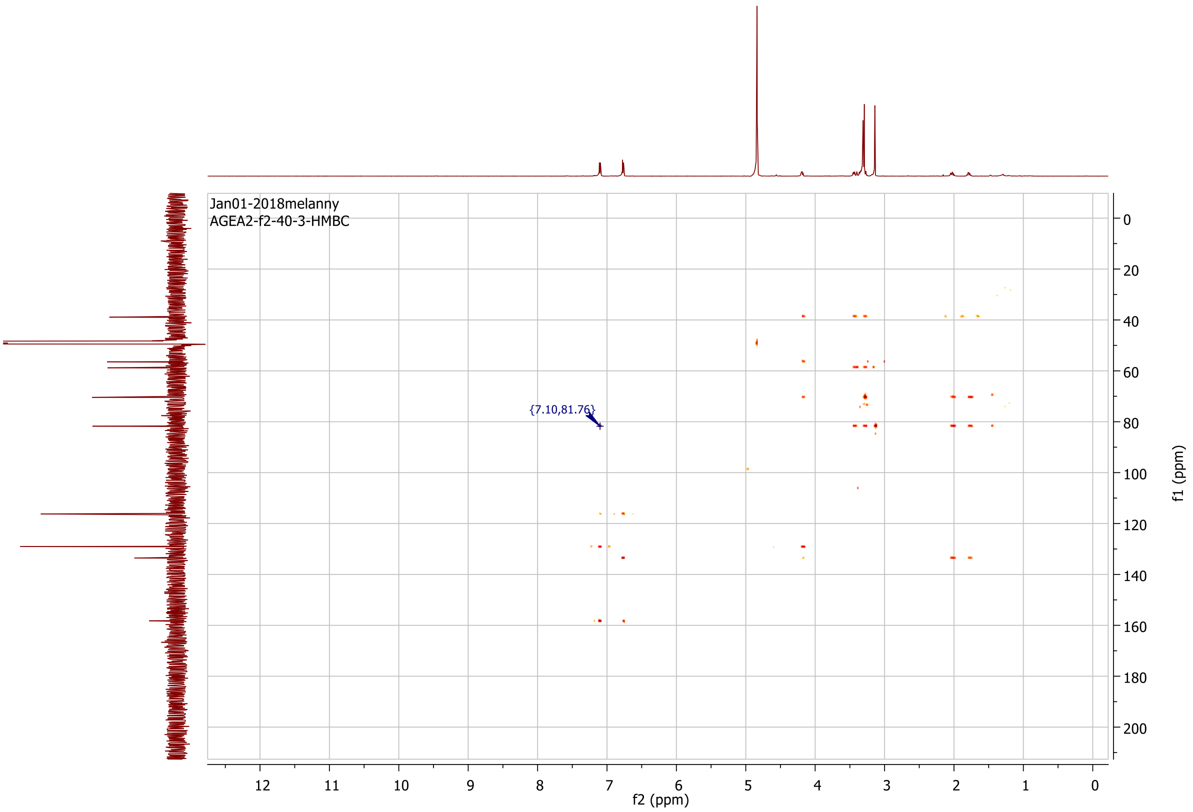

Figure S19 IR spectrum of 2

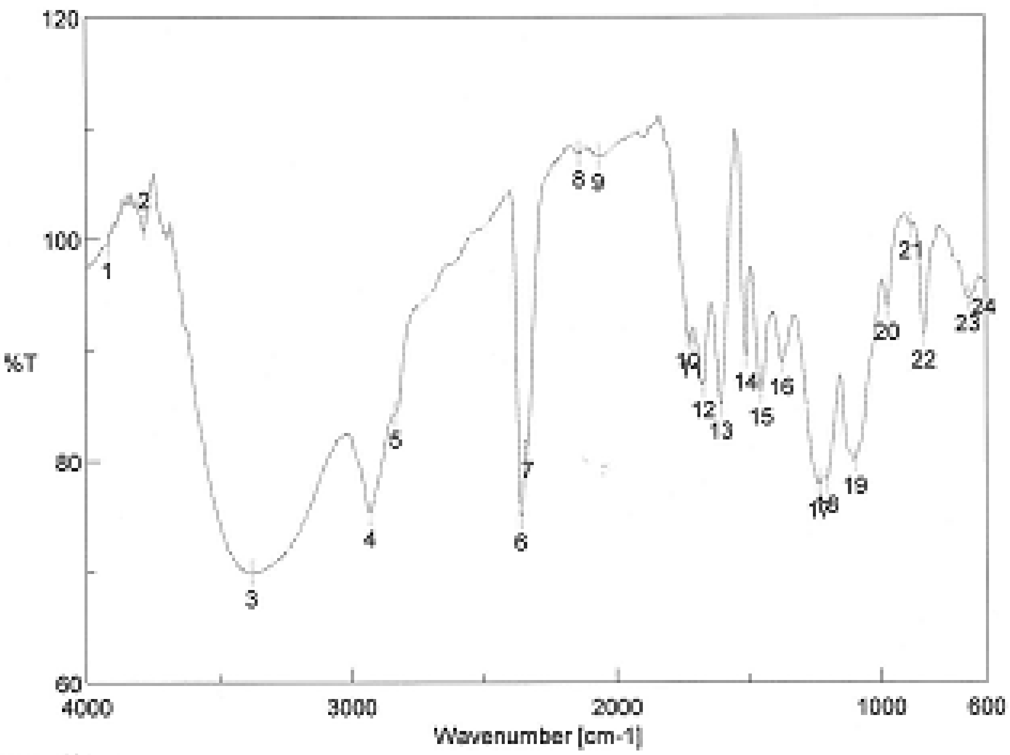

[コメント情報]
試料名
コメント
測定者
所属
会社

[データ情報]		[測定情報]	
作成日時	2018/08/20 13:20	機種名	FT/IR-4600typeA
		シリアル番号	D015461788
データタイプ	等間隔データ		
横軸	Wavenumber [cm-1]	測定日時	2018/08/20 12:41
縦軸	%T		
スタート	599.753 cm-1	光源	標準光源
エンド	7800.65 cm-1	検出器	TGS
データ間隔	0.964233 cm-1	積算回数	10
データ数	7469	分解	4 cm-1
		ゼロフィリング	On
		アポダイゼーション	Cosine
		ゲイン	Auto (2)
		アパーチャー	Auto (7.1 mm)
		スキャンスピード	Auto (2 mm/sec)
		フィルタ	Auto (30000 Hz)

[ピーク検出結果]

No.	位置	強度	No.	位置	強度
1	3918.64	99.4911	2	3777.87	101.081
3	3378.67	69.9199	4	2930.31	75.3527
5	2833.88	84.2422	6	2360.44	75.0184
7	2335.37	81.525	8	2138.67	107.656
9	2066.35	107.394	10	1729.83	91.3333
11	1718.26	90.6165	12	1671.02	86.9529
13	1604.48	85.0544	14	1509.98	89.4425

Figure S20 HR-ESI-MS of 3

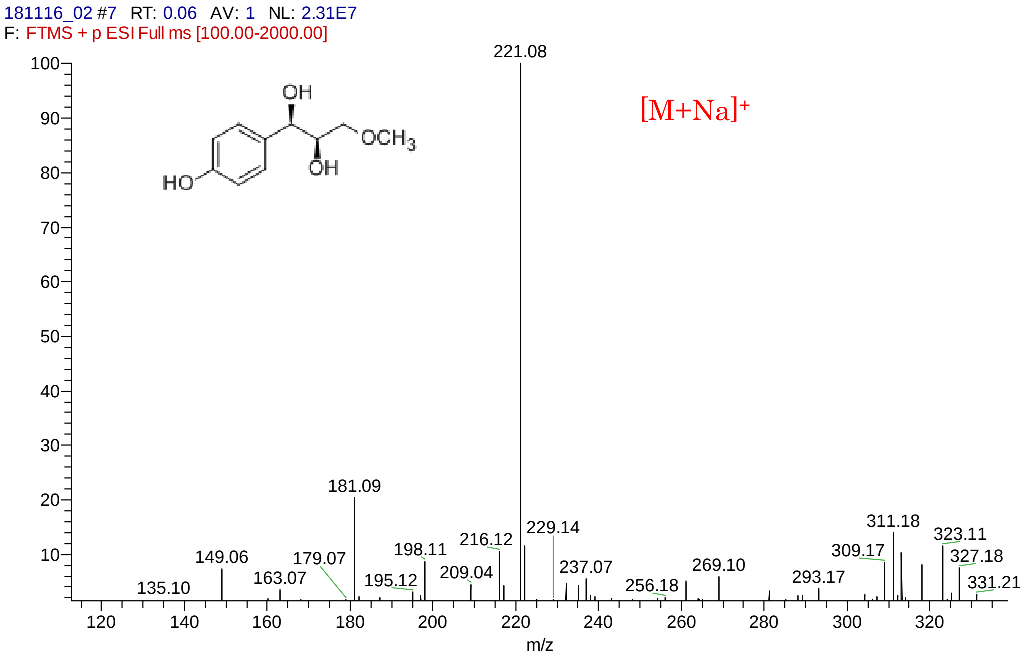

Elemental composition search on mass 221.08

m/z= 216.08-226.08

m/z	Theo. Mass	Delta (ppm)	RDB equiv.	Composition
221.0781	221.0784	-1.58	3.5	$C_{10}H_{14}O_4Na$

Figure S21 ^{1}H NMR spectrum of 3

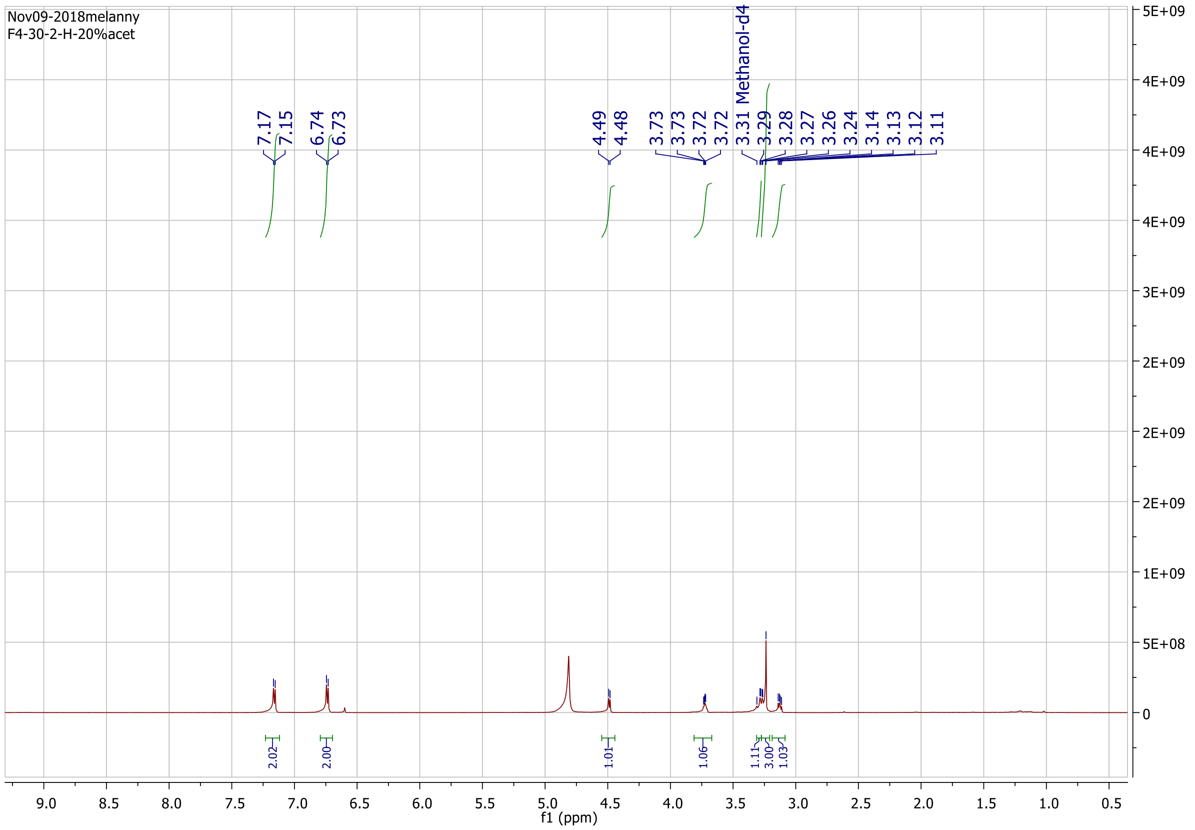

Figure S22 ^{13}C NMR spectrum of 3

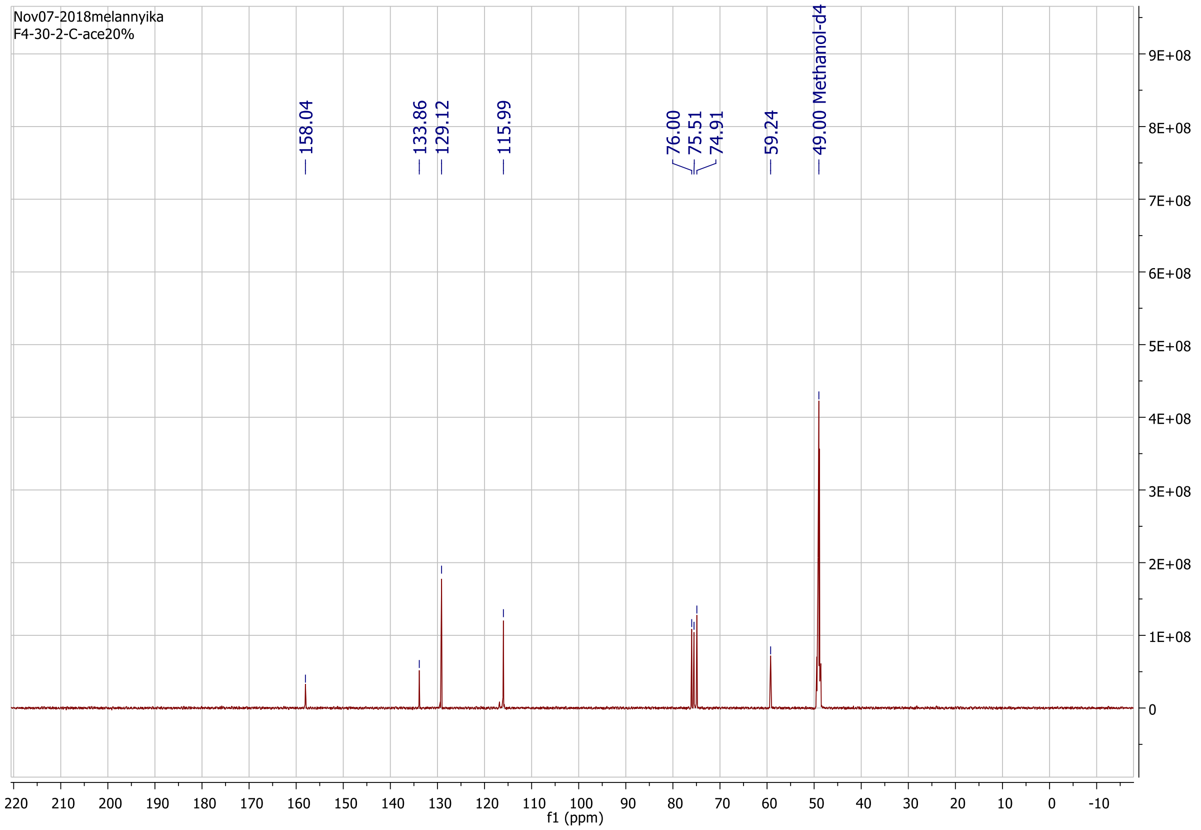

Figure S23 DEPT 135 spectrum of 3

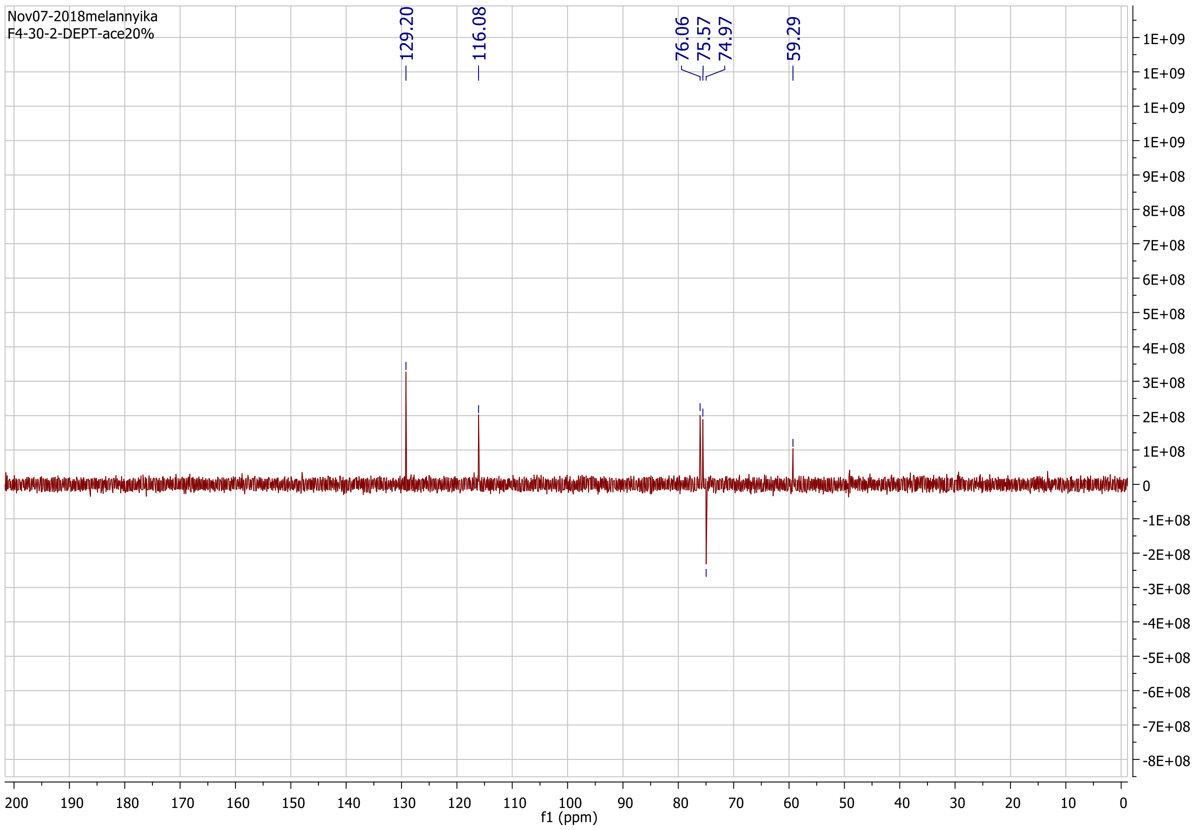

Figure S24 COSY spectrum of 3

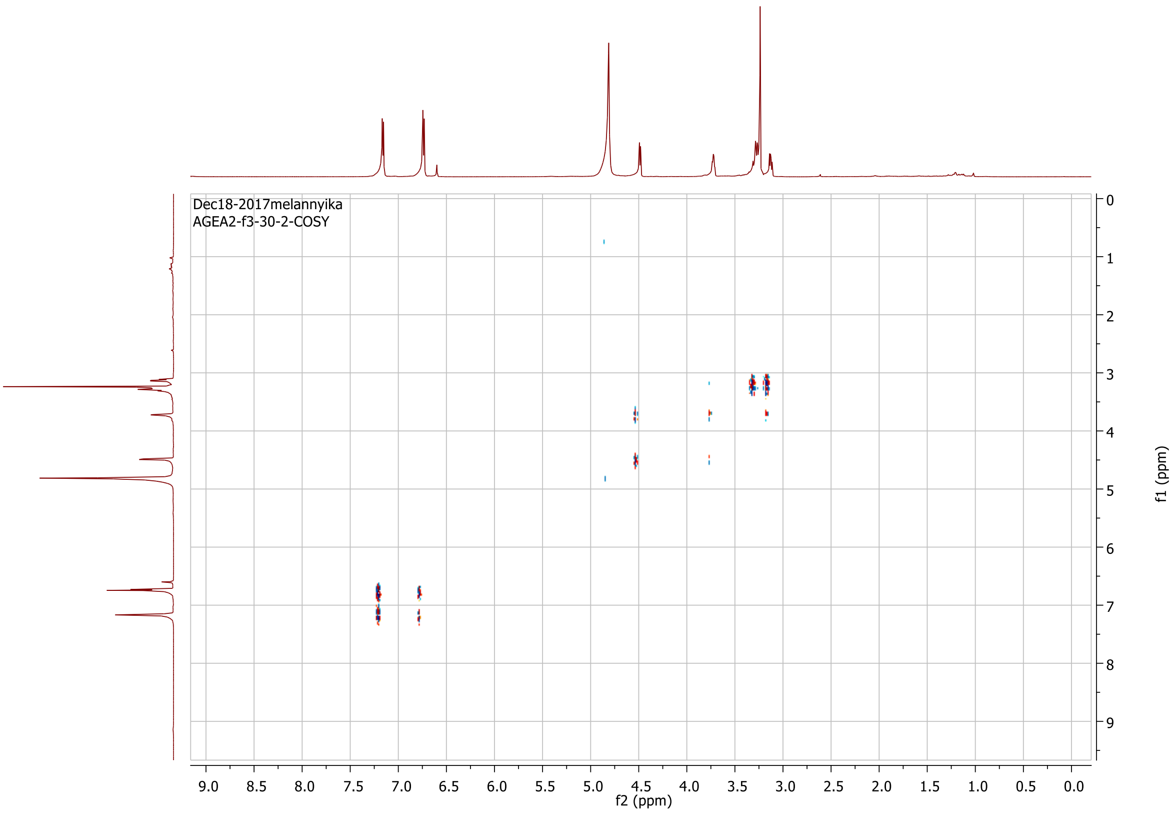

Figure S25 HSQC spectrum of 3

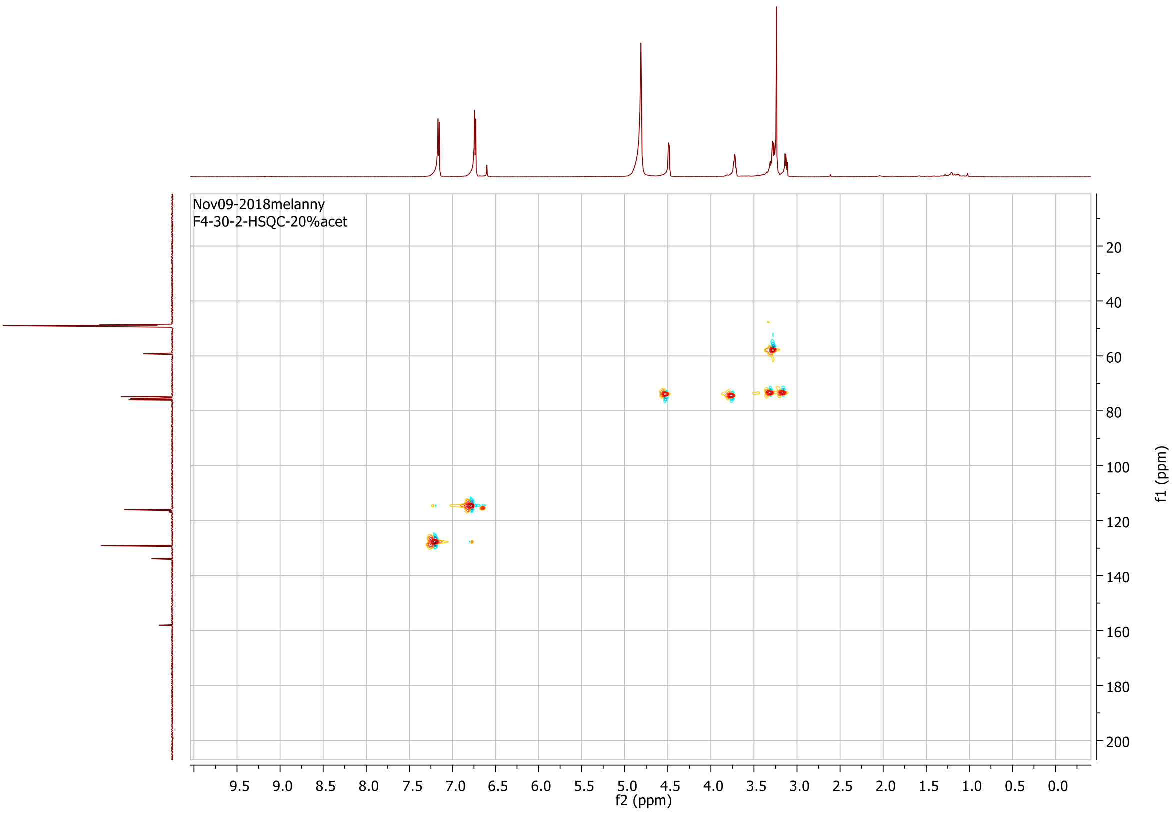

Figure S26 HMBC spectrum of 3

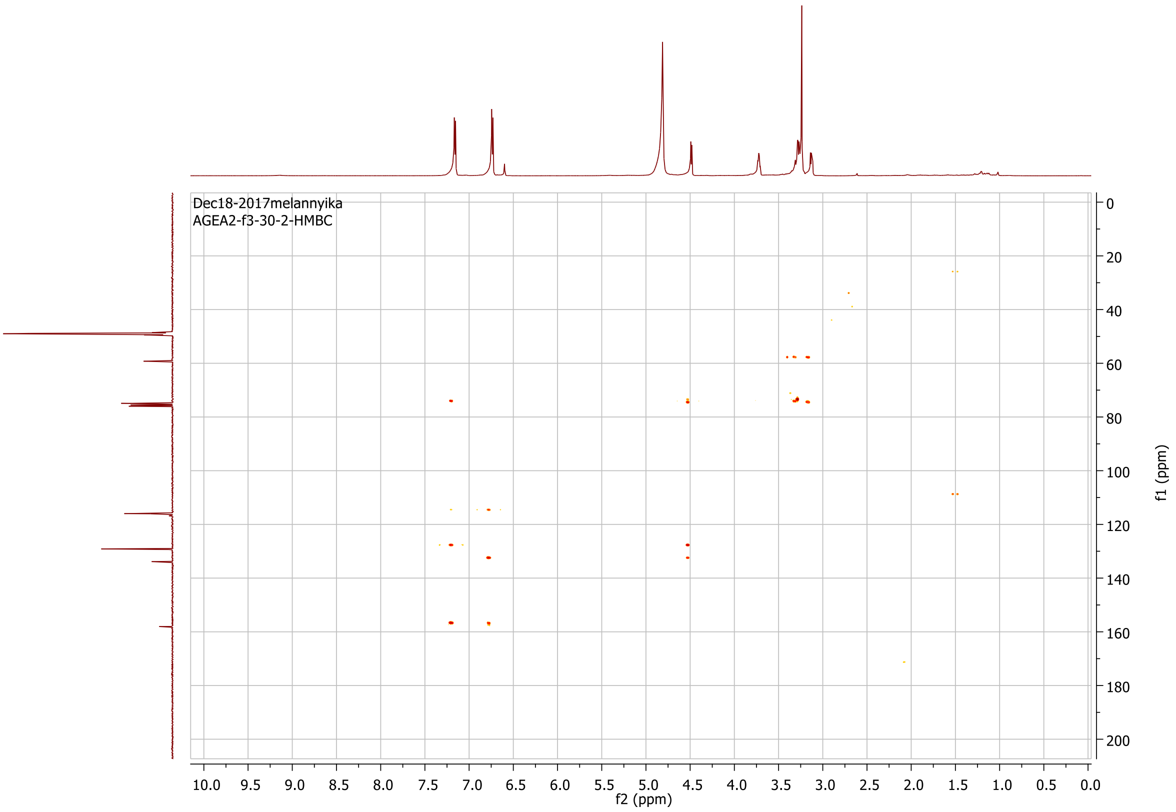

Figure S27 IR spectrum of 3

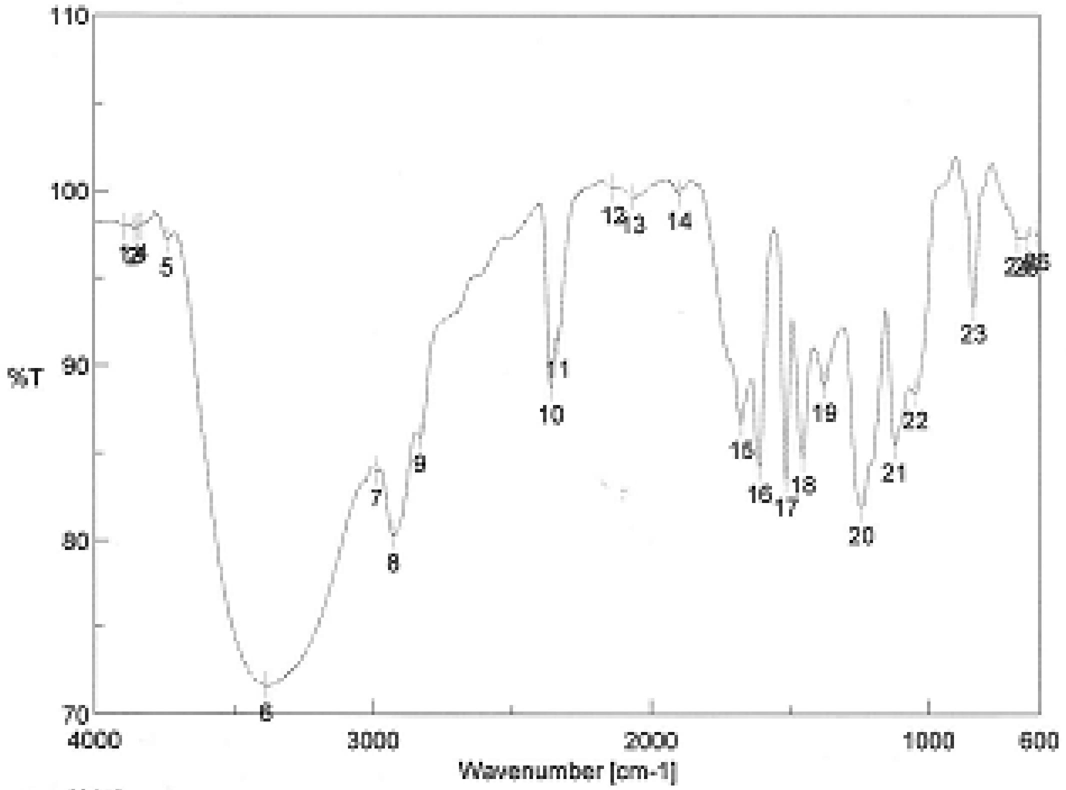

[コメント情報]

試料名	
コメント	
測定者	
所属	
会社	HU

[データ情報]

作成日時	2018/08/20 13:16
データタイプ	等間隔データ
横軸	Wavenumber [cm-1]
縦軸	%T
スタート	599.753 cm-1
エンド	7800.65 cm-1
データ間隔	0.964233 cm-1
データ数	7469

[測定情報]

機種名	FT/IR-4600typeA
シリアル番号	D015461786
測定日時	2018/08/20 11:37
光源	標準光源
検出器	TGS
積算回数	10
分解	4 cm-1
ゼロフィリング	On
アポダイゼーション	Cosine
ゲイン	Auto (2)
アパーチャー	Auto (7.1 mm)
スキャンスピード	Auto (2 mm/sec)
フィルタ	Auto (30000 Hz)

[ピーク検出結果]

No.	位置	強度	No.	位置	強度
1	3892.61	97.8906	2	3861.76	97.76
3	3844.4	97.8238	4	3829.94	97.9445
5	3740.26	97.1194	6	3383.5	71.6524
7	2987.2	83.9784	8	2926.45	80.2416
9	2830.99	85.9292	10	2360.44	88.7435
11	2334.41	91.3363	12	2134.81	100.125
13	2064.42	99.5811	14	1897.61	99.8014

Figure S28 HR-ESI-MS of 4

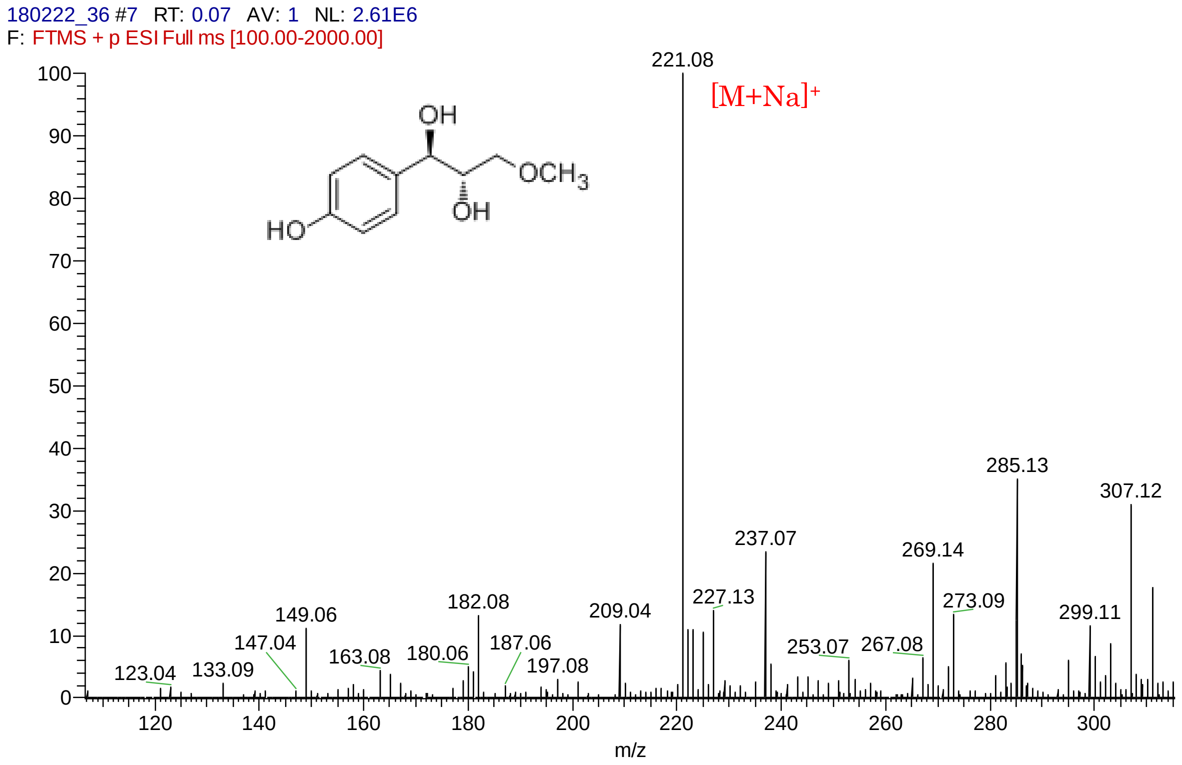

Elemental composition search on mass 221.08

m/z= 216.08-226.08

m/z	Theo. Mass	Delta (ppm)	RDB equiv.	Composition
221.0781	221.0784	-1.58	3.5	$C_{10} H_{14} O_4 Na$

Figure S29 ^{1}H NMR spectrum of 4

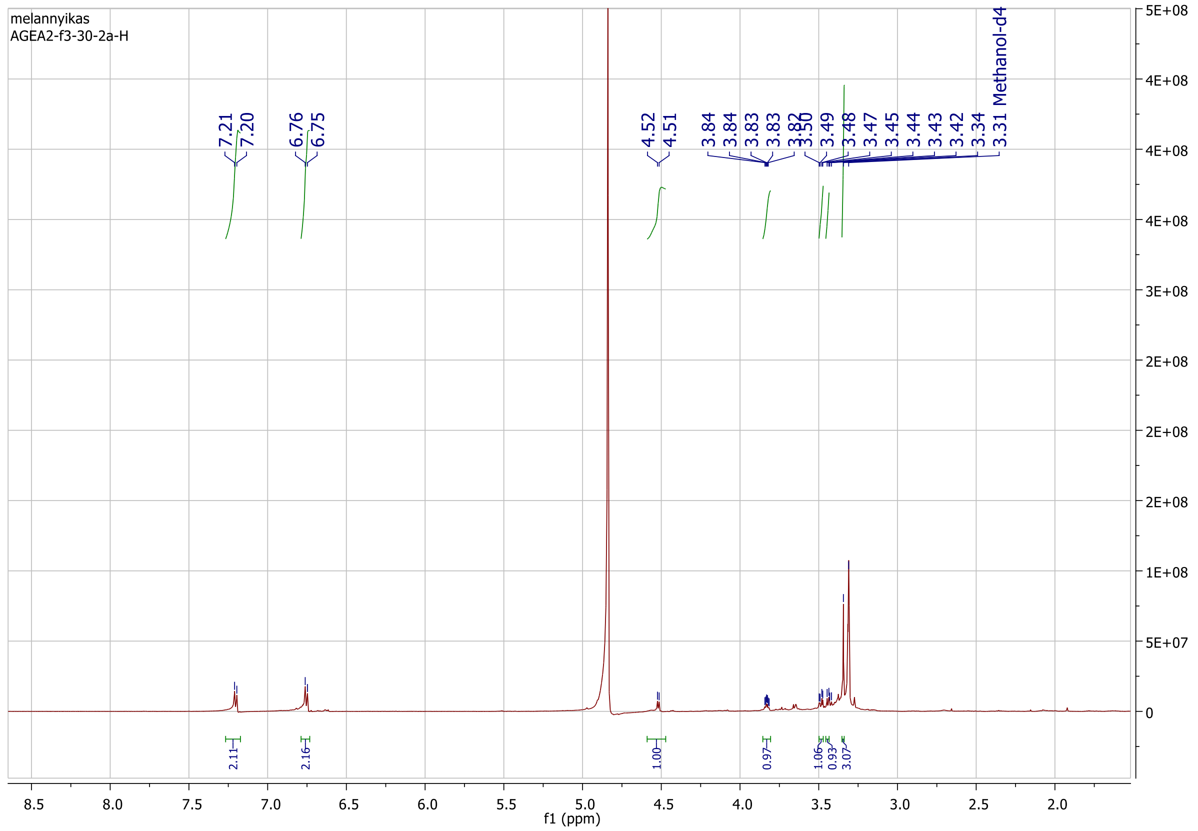

Figure S30 ^13^C NMR spectrum of 4

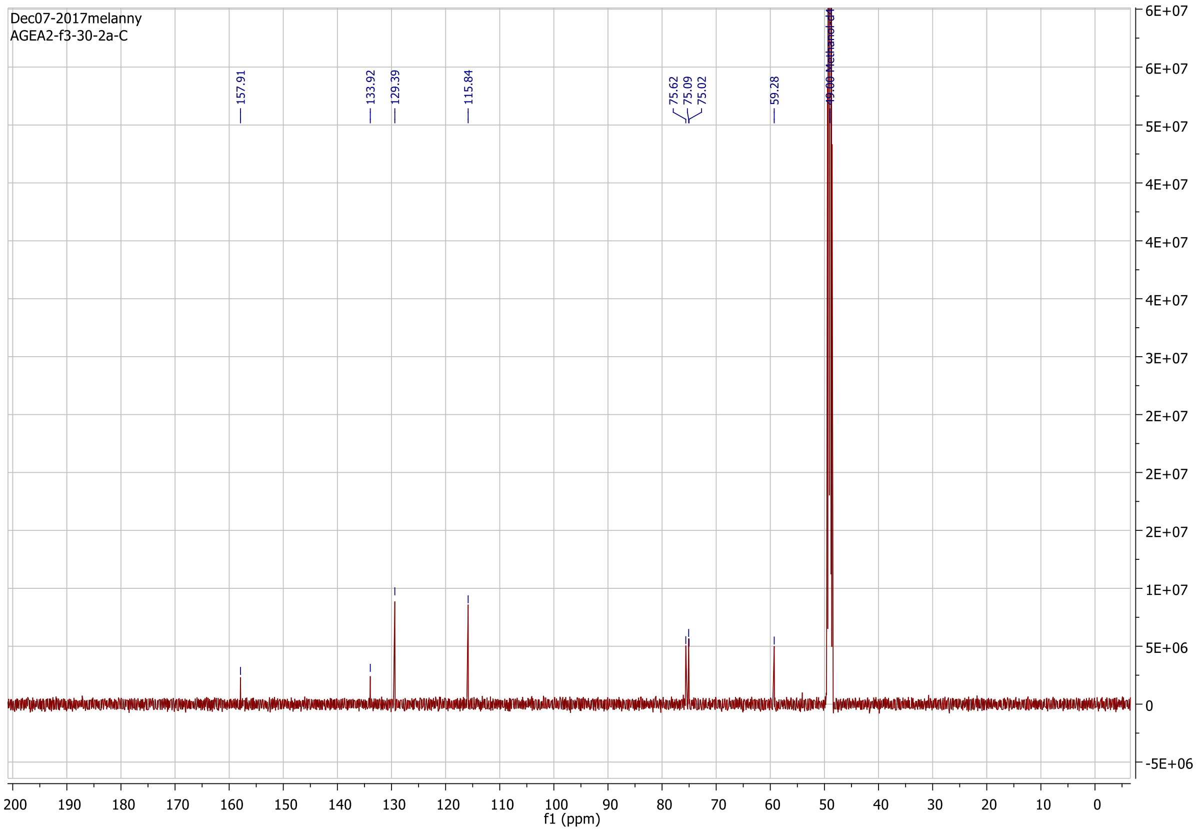

Figure S31 DEPT 135 spectrum of 4

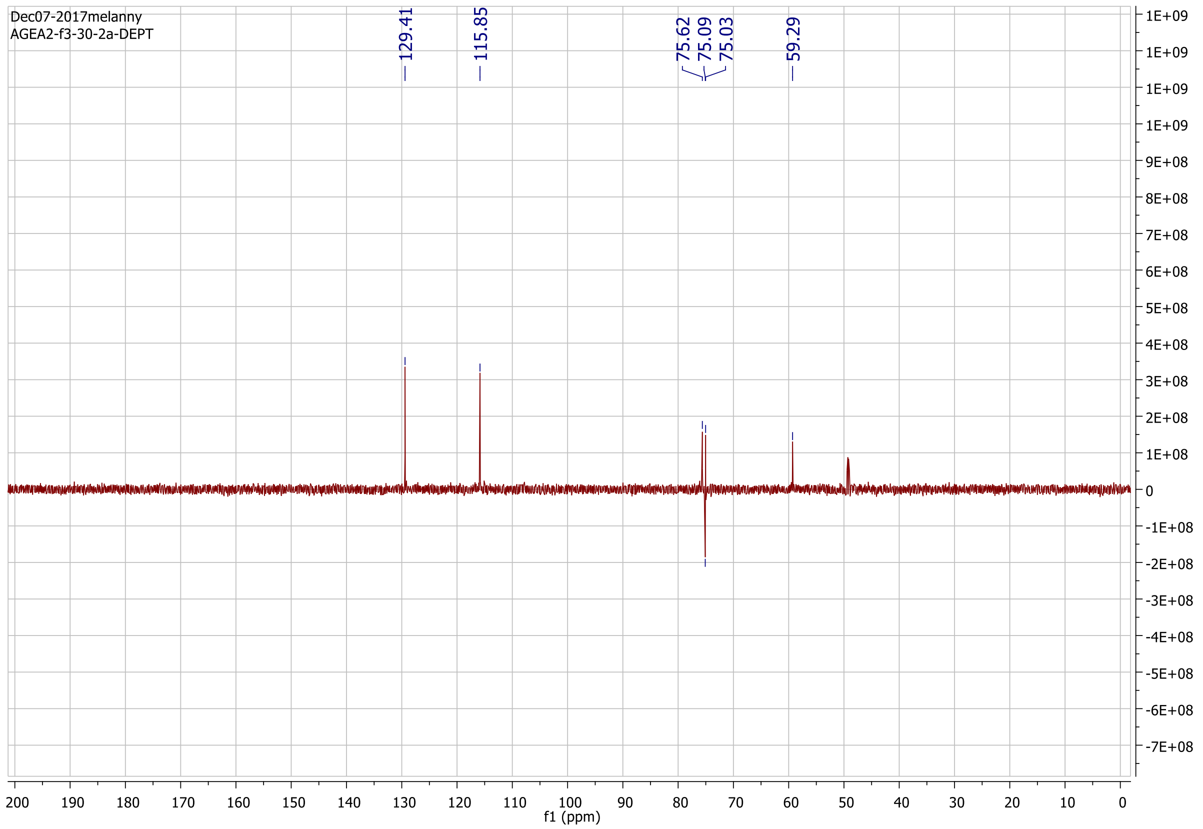

Figure S32 **COSY spectrum of 4**

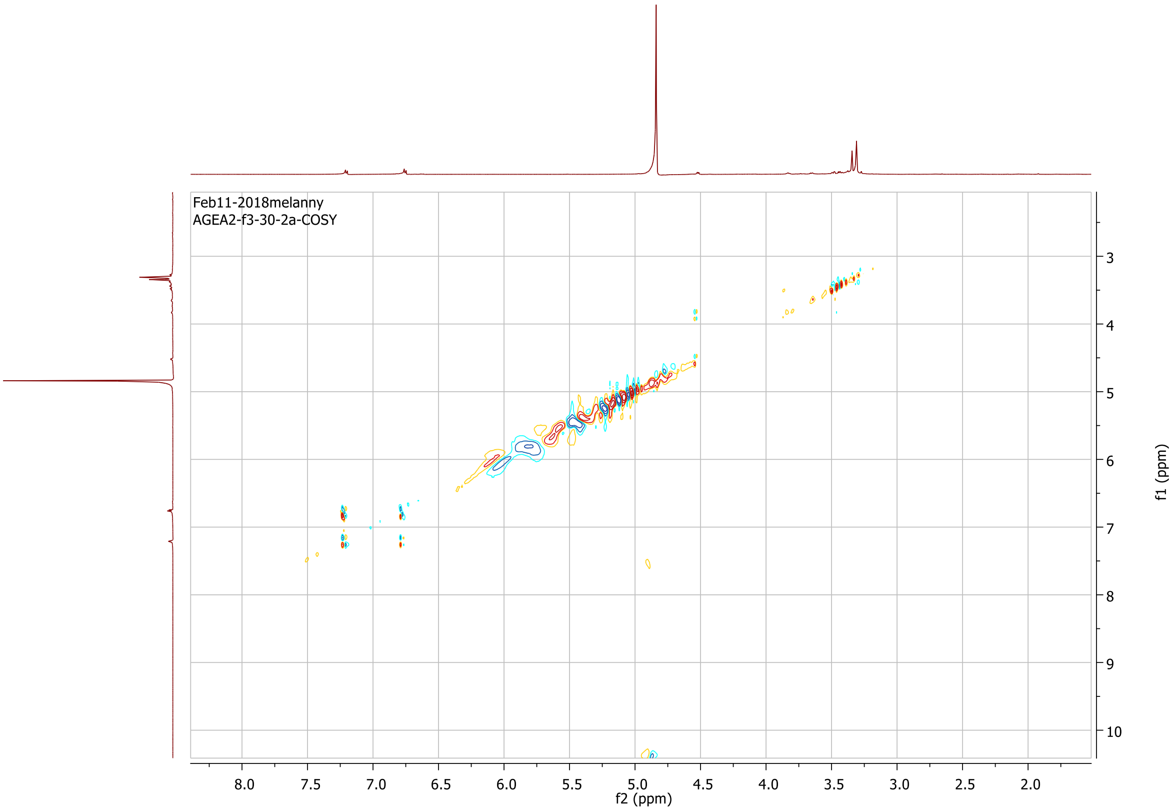

Figure S33   HSQC spectrum of 4

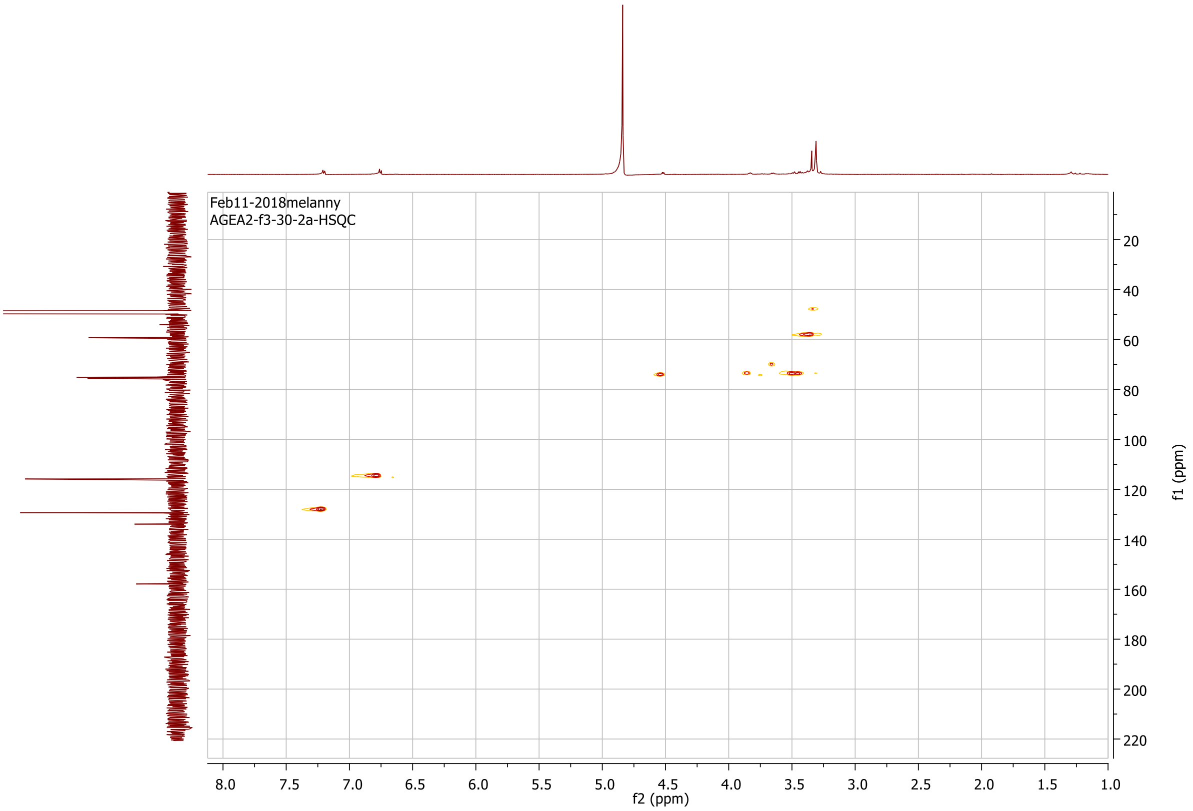

Figure S34 HMBC spectrum of 4

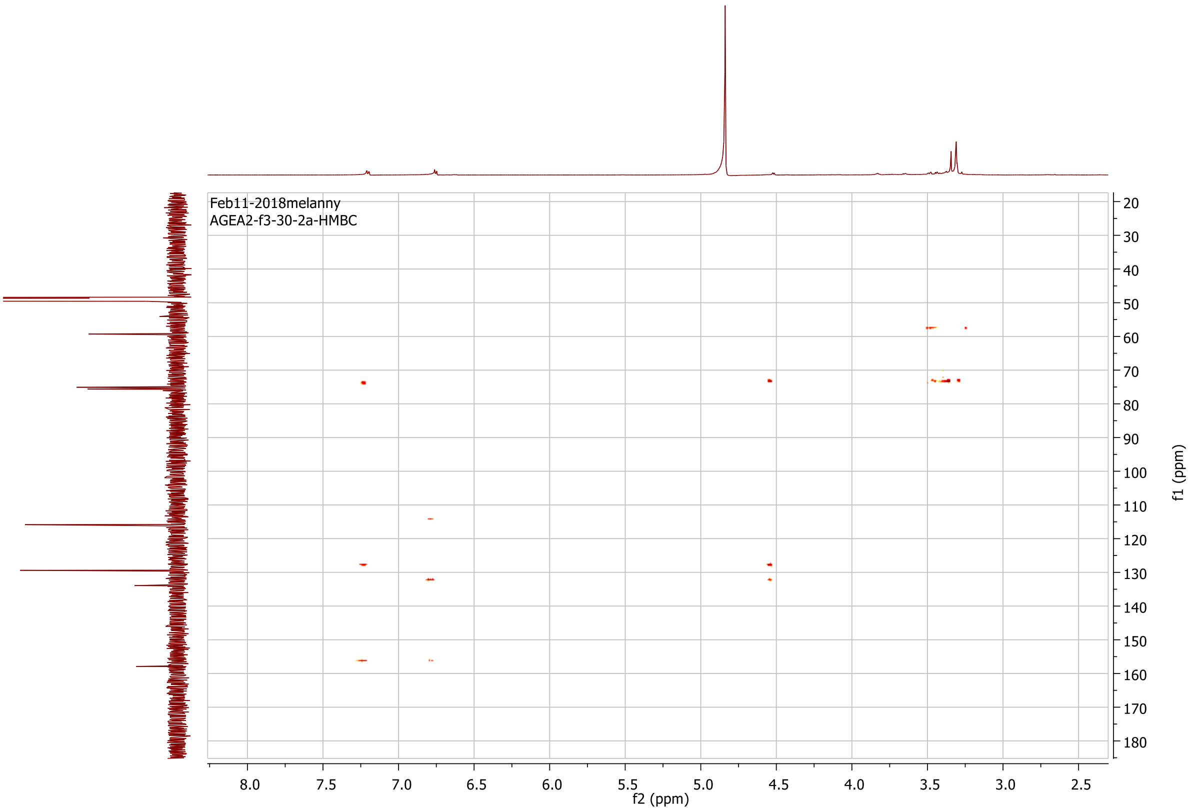

Figure S35 IR spectrum of 4

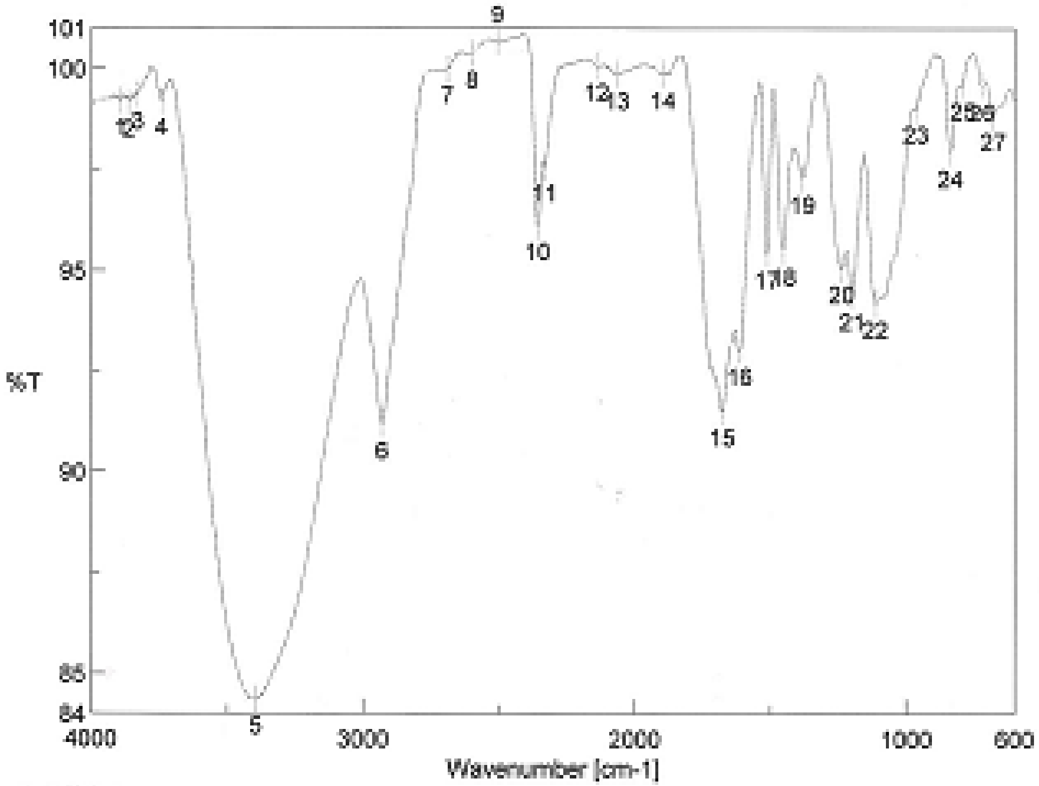

[コメント情報]
試料名
コメント
測定者
所属
会社 HU

[データ情報]		[測定情報]	
作成日時	2018/08/20 13:25	機種名	FT/IR-4600typeA
		シリアル番号	D015461786
データタイプ	等間隔データ		
横軸	Wavenumber [cm-1]	測定日時	2018/08/20 11:21
縦軸	%T		
スタート	599.753 cm-1	光源	標準光源
エンド	7800.65 cm-1	検出器	TGS
データ間隔	0.964233 cm-1	積算回数	10
データ数	7469	分解	4 cm-1
		ゼロフィリング	On
		アポダイゼーション	Cosine
		ゲイン	Auto (2)
		アパーチャー	Auto (7.1 mm)
		スキャンスピード	Auto (2 mm/sec)
		フィルタ	Auto (30000 Hz)

[ピーク検出結果]

No.	位置	強度	No.	位置	強度
1	3892.61	99.1829	2	3861.76	99.146
3	3829.94	99.3463	4	3742.19	99.196
5	3394.1	84.3837	6	2930.31	91.1735
7	2691.18	100.008	8	2600.54	100.387
9	2507.01	100.673	10	2360.44	96.1302
11	2333.45	97.5762	12	2142.53	100.076
13	2067.32	99.8592	14	1897.61	99.8663

Figure S36 HR-ESI-MS of 5

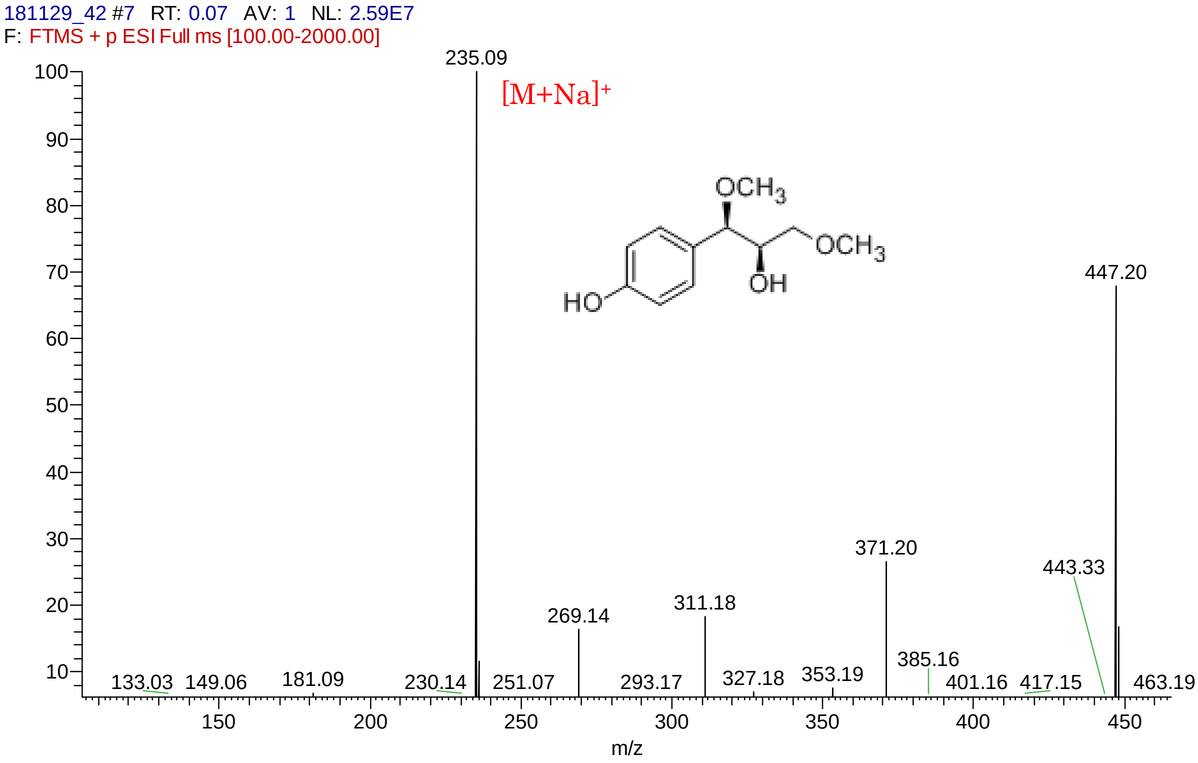

Elemental composition search on mass 235.09

m/z= 230.09-240.09

m/z	Theo. Mass	Delta (ppm)	RDB equiv.	Composition
235.0939	235.0941	-0.89	3.5	$C_{11}H_{16}O_4Na$

Figure S37 ^{1}H NMR spectrum of 5

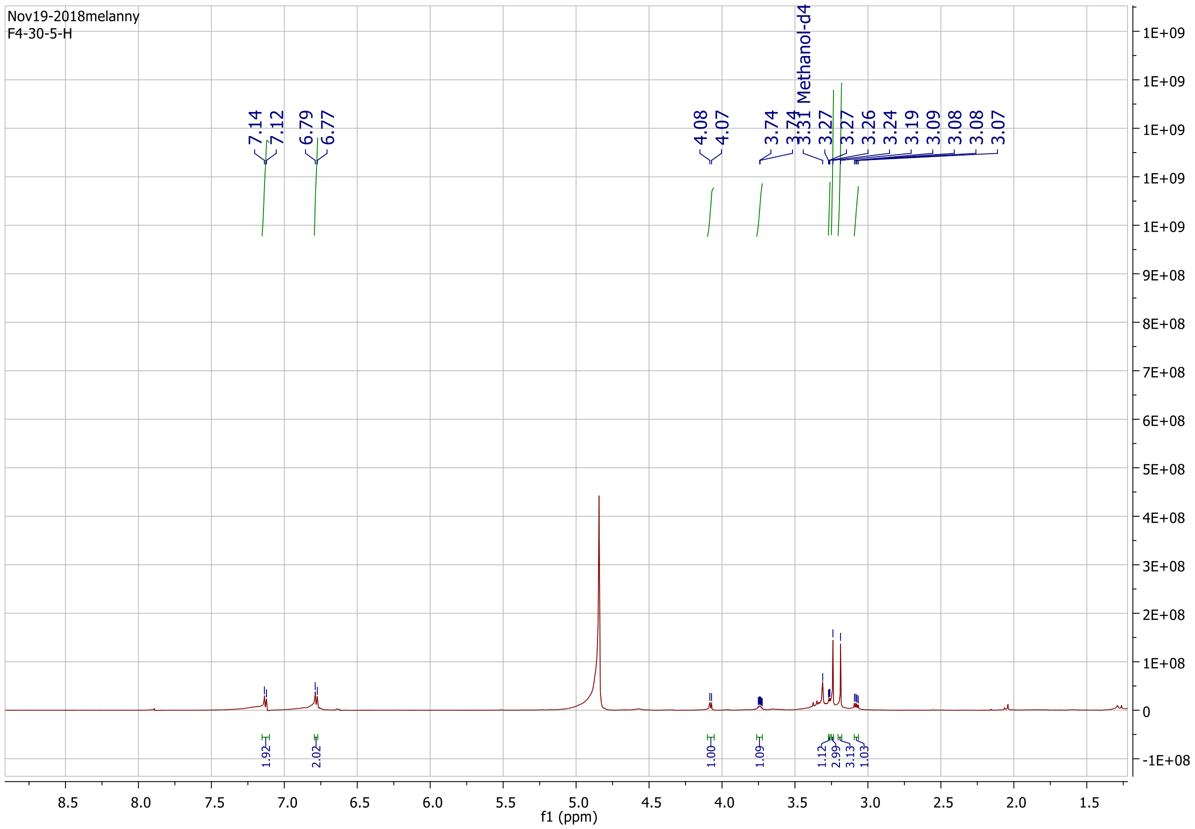

Figure S38 ^{13}C NMR spectrum of 5

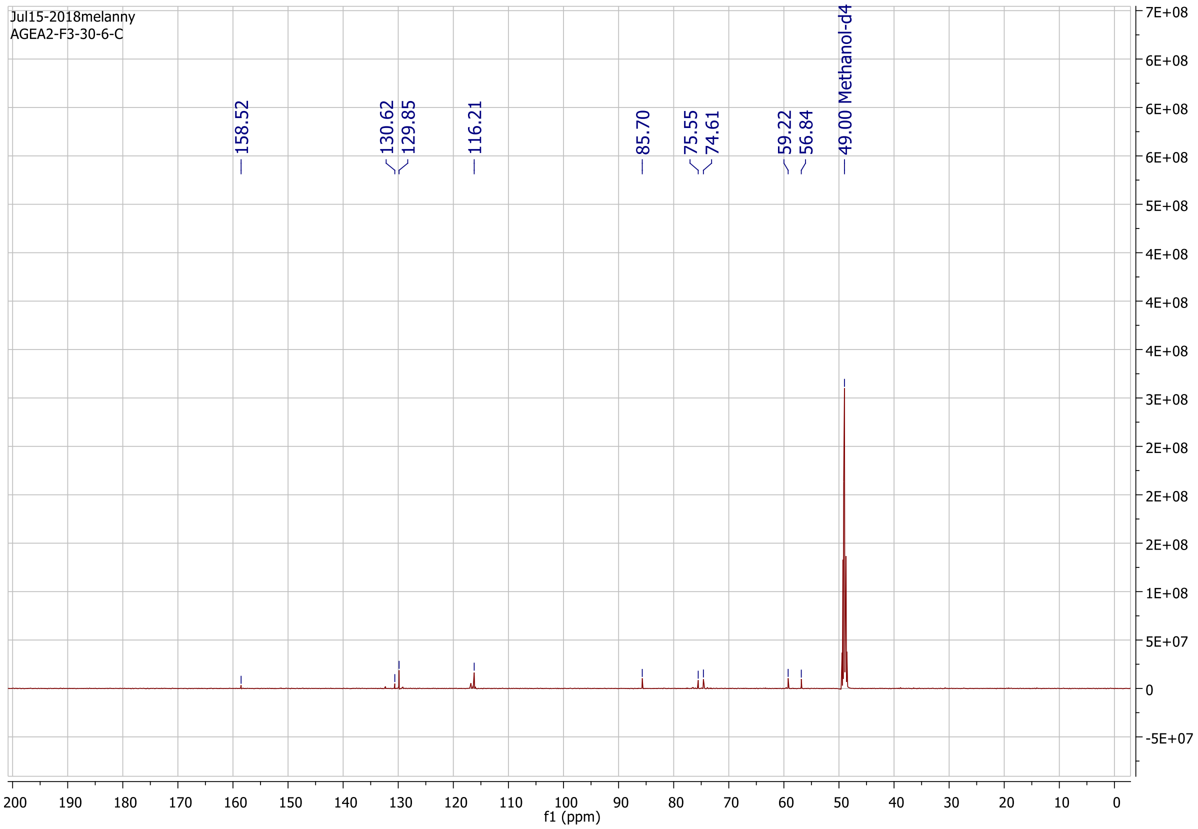

Figure S39 DEPT 135 spectrum of 5

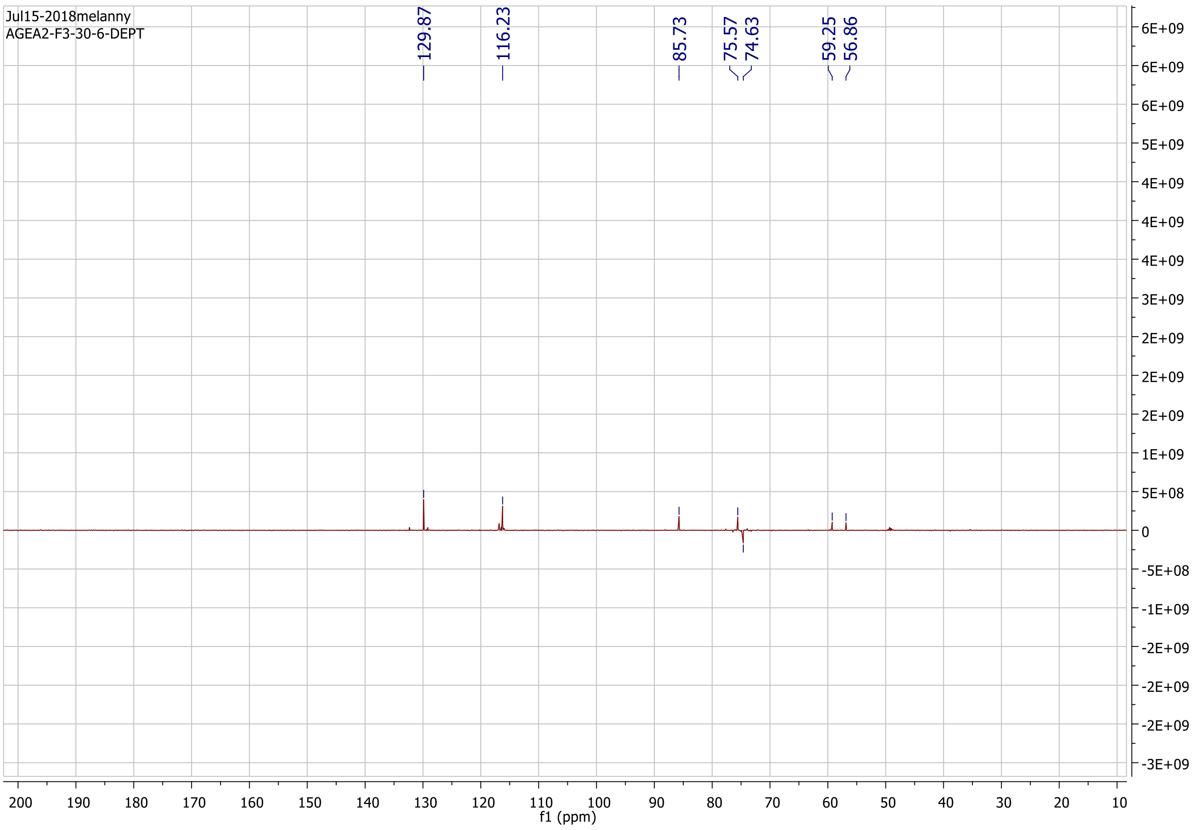

Figure S40 COSY spectrum of 5

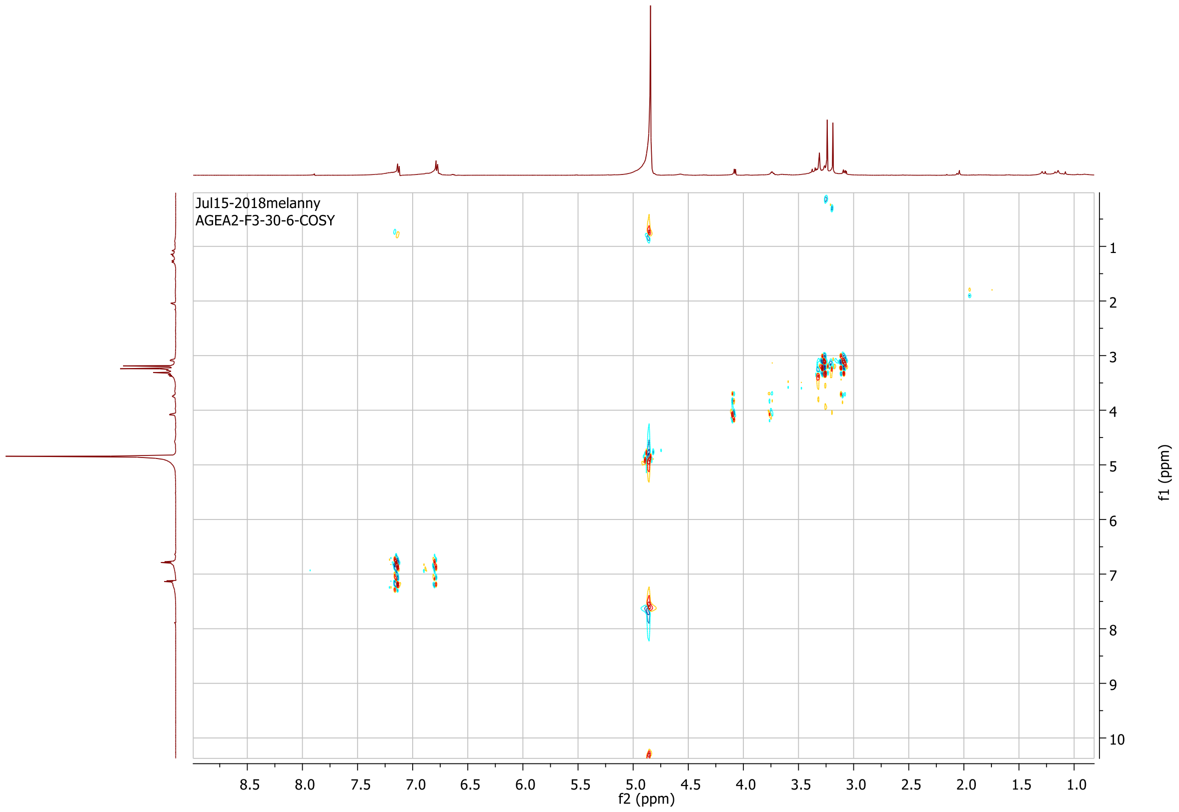

Figure S41 HSQC spectrum of 5

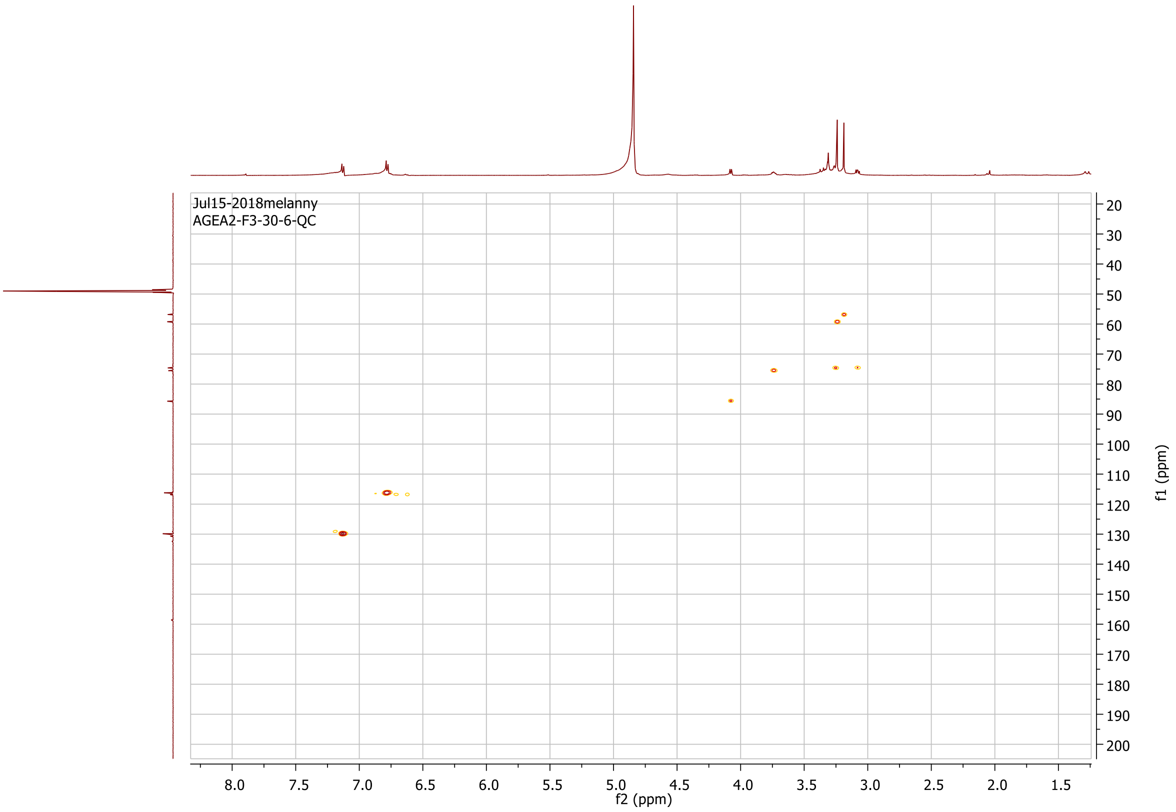

Figure S42 HMBC spectrum of 5

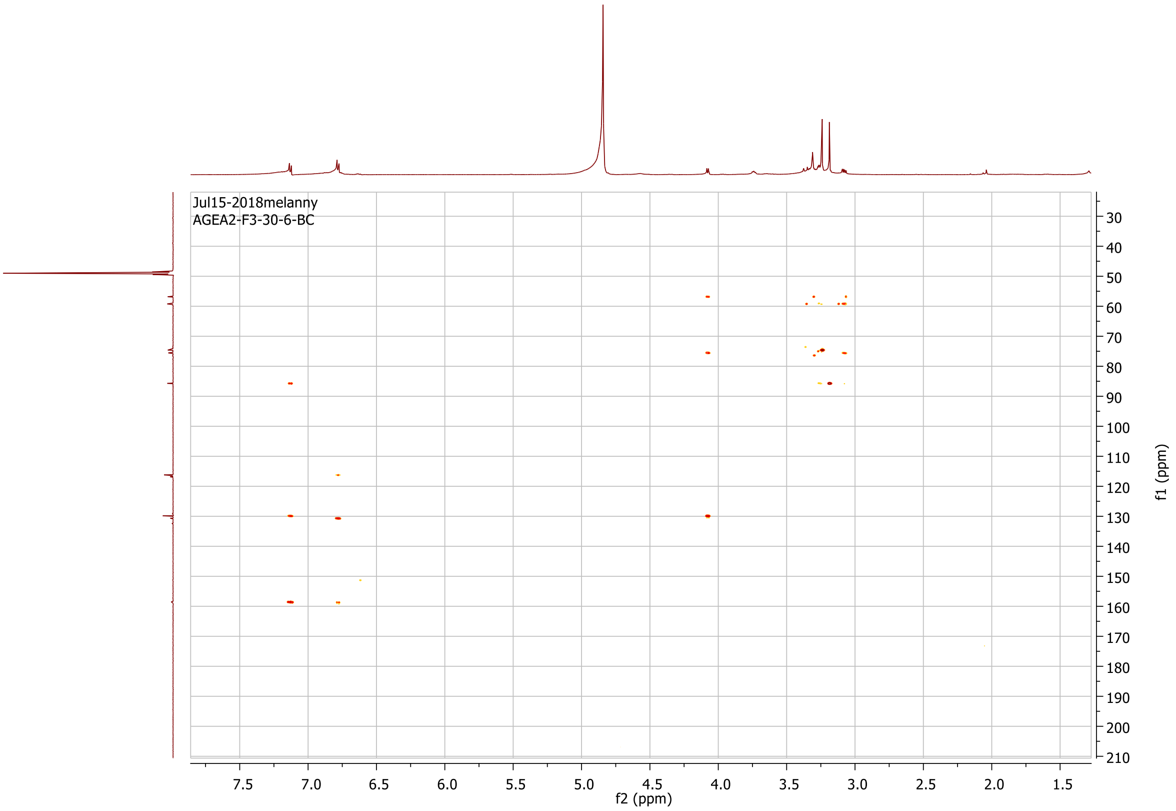

Figure S43 IR spectrum of 5

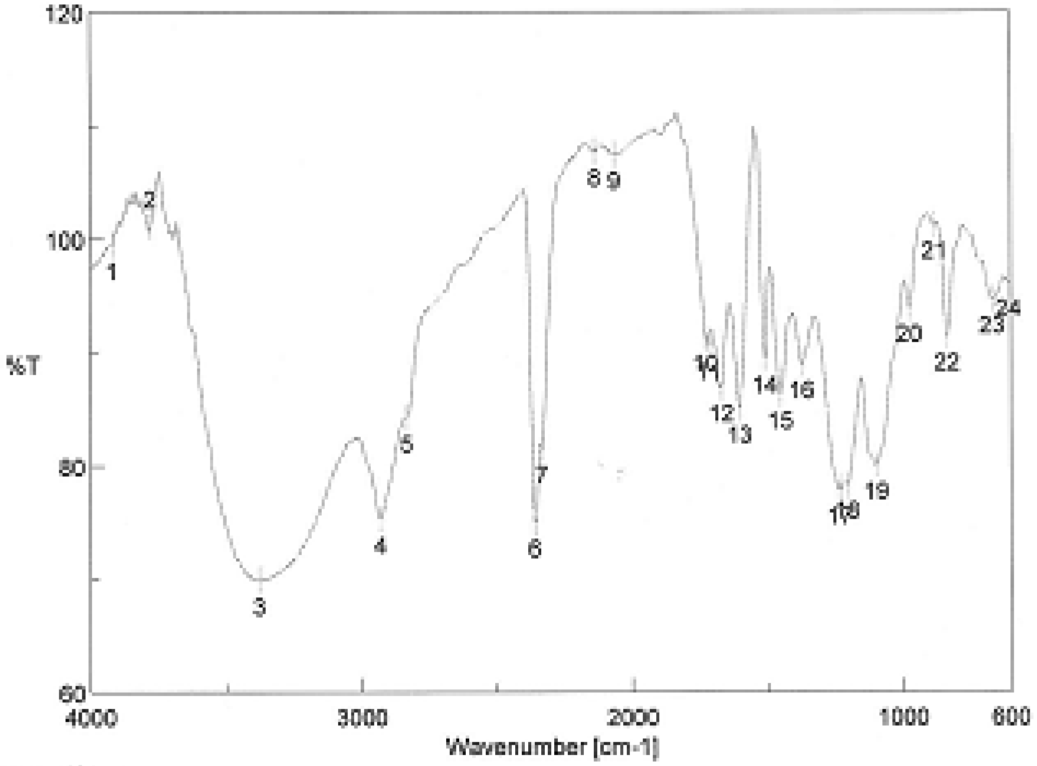

[コメント情報]
試料名
コメント
測定者
所属
会社 HU

[データ情報]		[測定情報]	
作成日時	2018/08/20 13:20	機種名	FT/IR-4600typeA
		シリアル番号	D015461788
データタイプ	等間隔データ		
横軸	Wavenumber [cm-1]	測定日時	2018/08/20 12:41
縦軸	%T		
スタート	599.753 cm-1	光源	標準光源
エンド	7800.65 cm-1	検出器	TGS
データ間隔	0.964233 cm-1	積算回数	10
データ数	7469	分解	4 cm-1
		ゼロフィリング	On
		アポダイゼーション	Cosine
		ゲイン	Auto (2)
		アパーチャー	Auto (7.1 mm)
		スキャンスピード	Auto (2 mm/sec)
		フィルタ	Auto (30000 Hz)

[ピーク検出結果]

No.	位置	強度	No.	位置	強度
1	3918.64	99.4911	2	3777.87	101.081
3	3378.67	69.9199	4	2930.31	75.3527
5	2833.88	84.2422	6	2360.44	75.0184
7	2335.37	81.525	8	2138.67	107.656
9	2066.35	107.394	10	1729.83	91.3333
11	1718.26	90.6165	12	1671.02	86.9529
13	1604.48	85.0544	14	1509.98	89.4425

Figure S44 HR-ESI-MS of 6

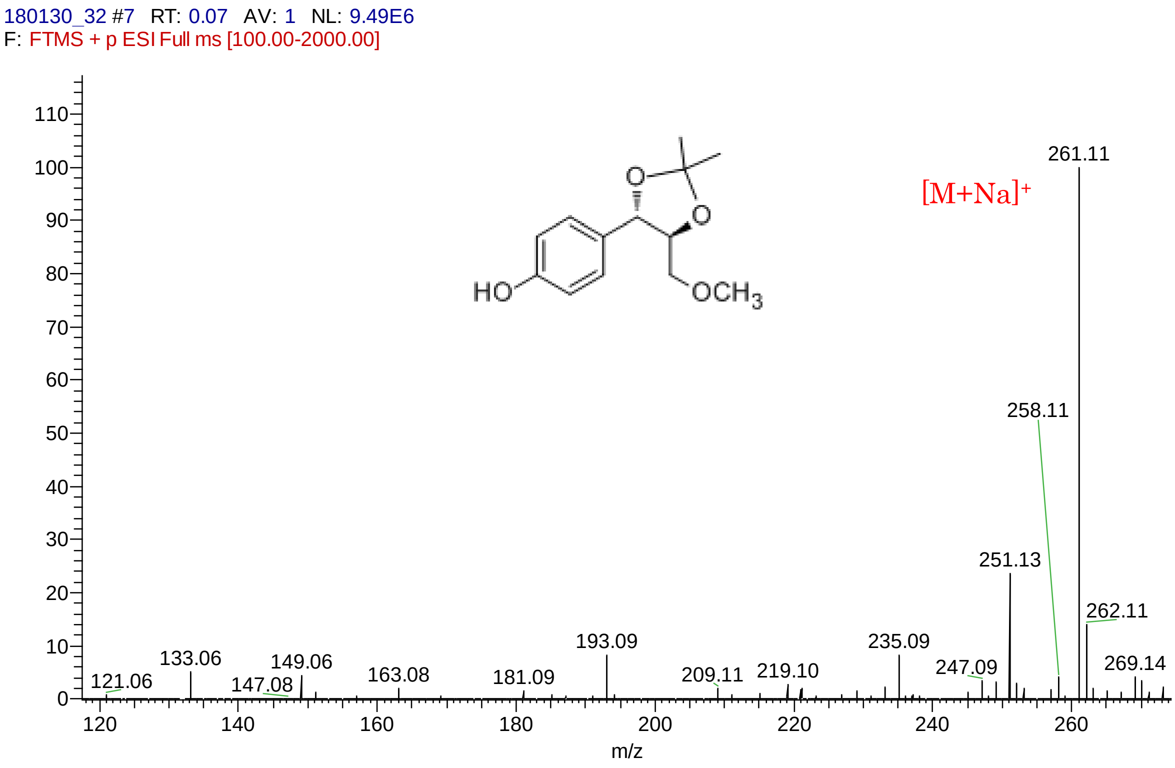

Elemental composition search on mass 261.11

m/z= 256.11-266.11

m/z	Theo. Mass	Delta (ppm)	RDB equiv.	Composition
261.1099	261.1097	0.50	4.5	$C_{13}H_{18}O_4Na$

Figure S45 ¹H NMR spectrum of 6

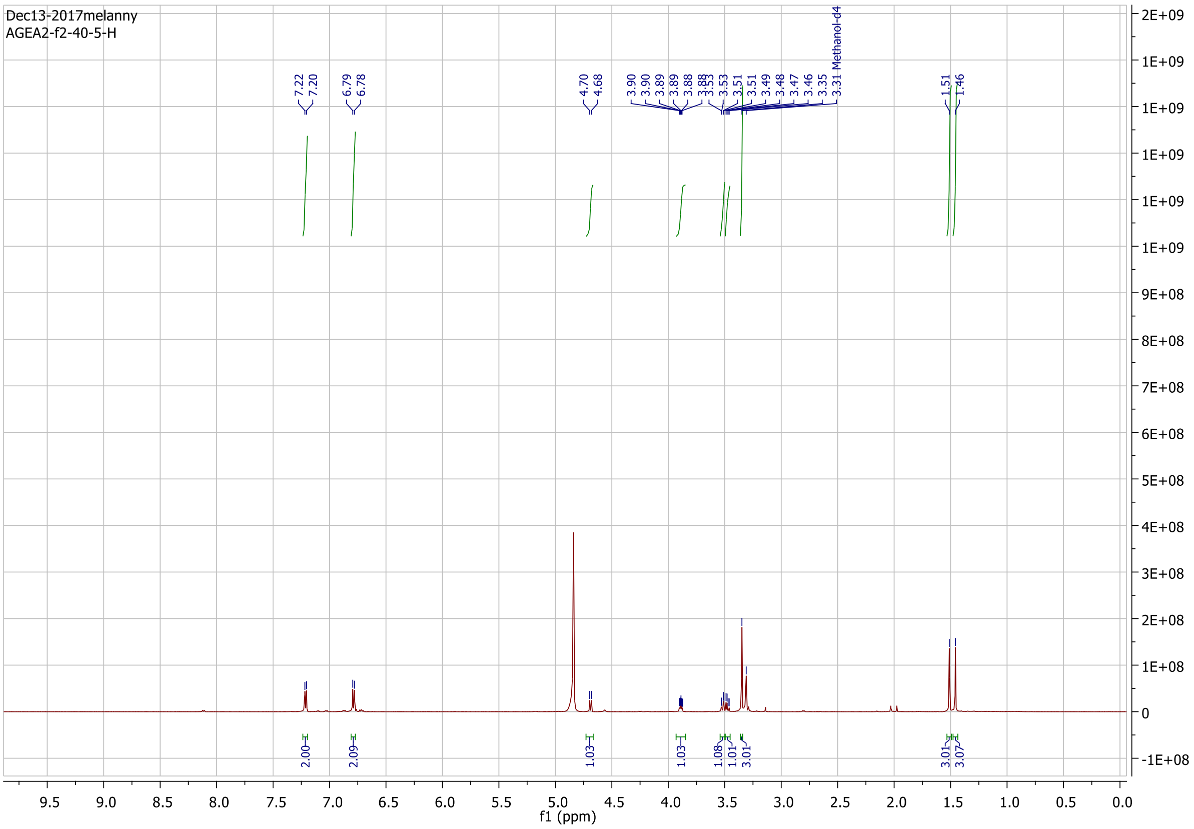

Figure S46 ^{13}C NMR spectrum of 6

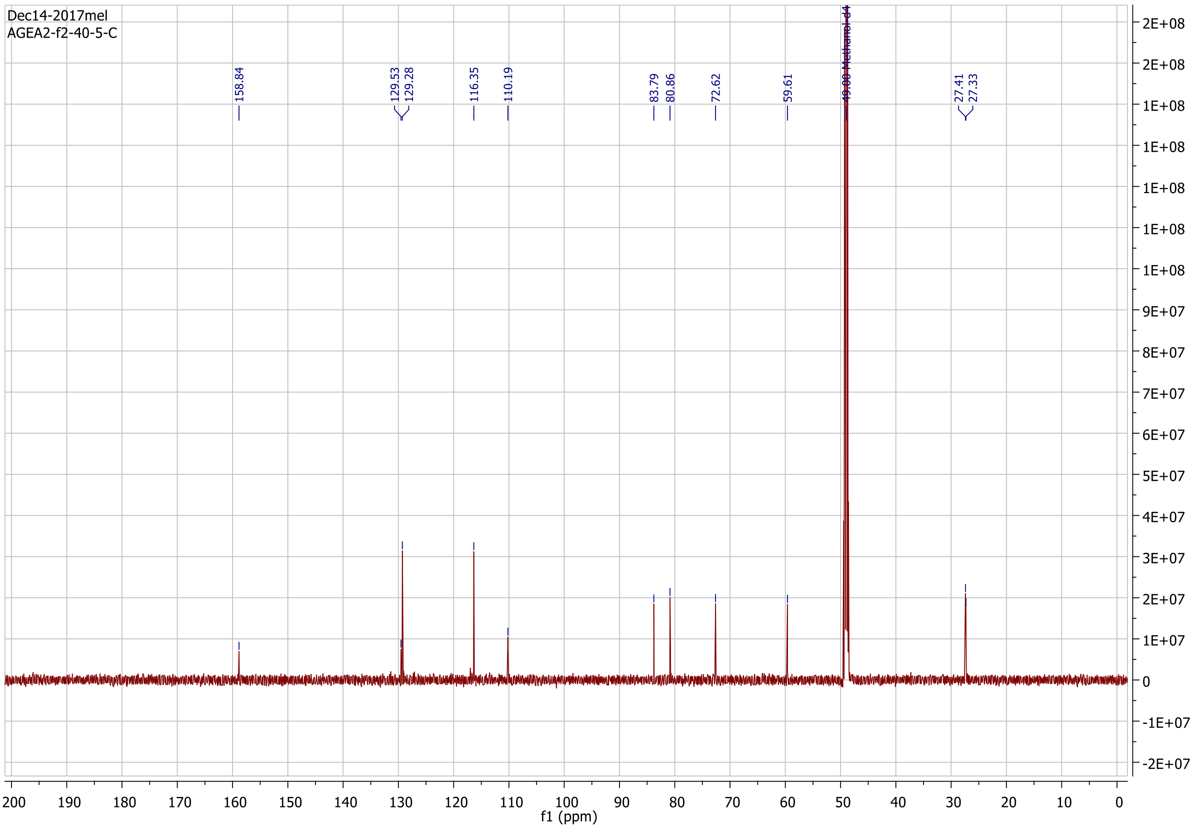

Figure S47 DEPT 135 spectrum of 6

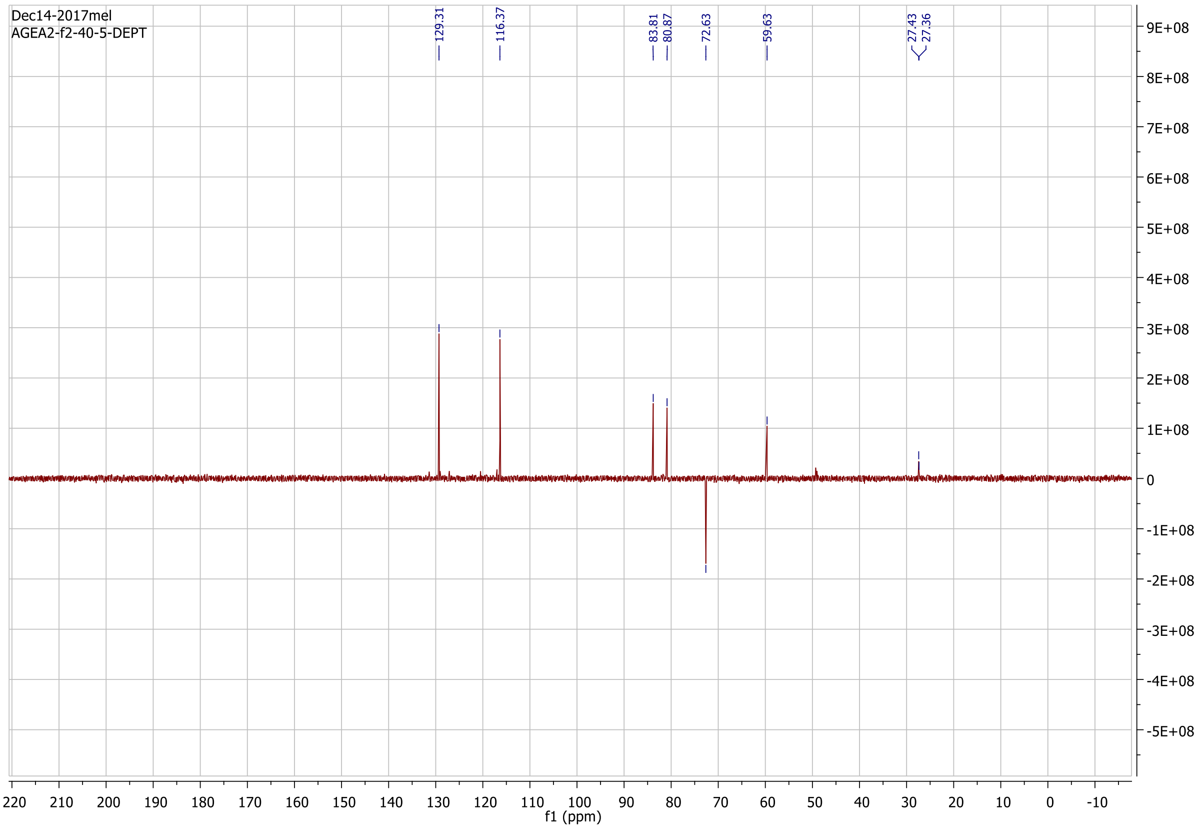

Figure S48 COSY spectrum of 6

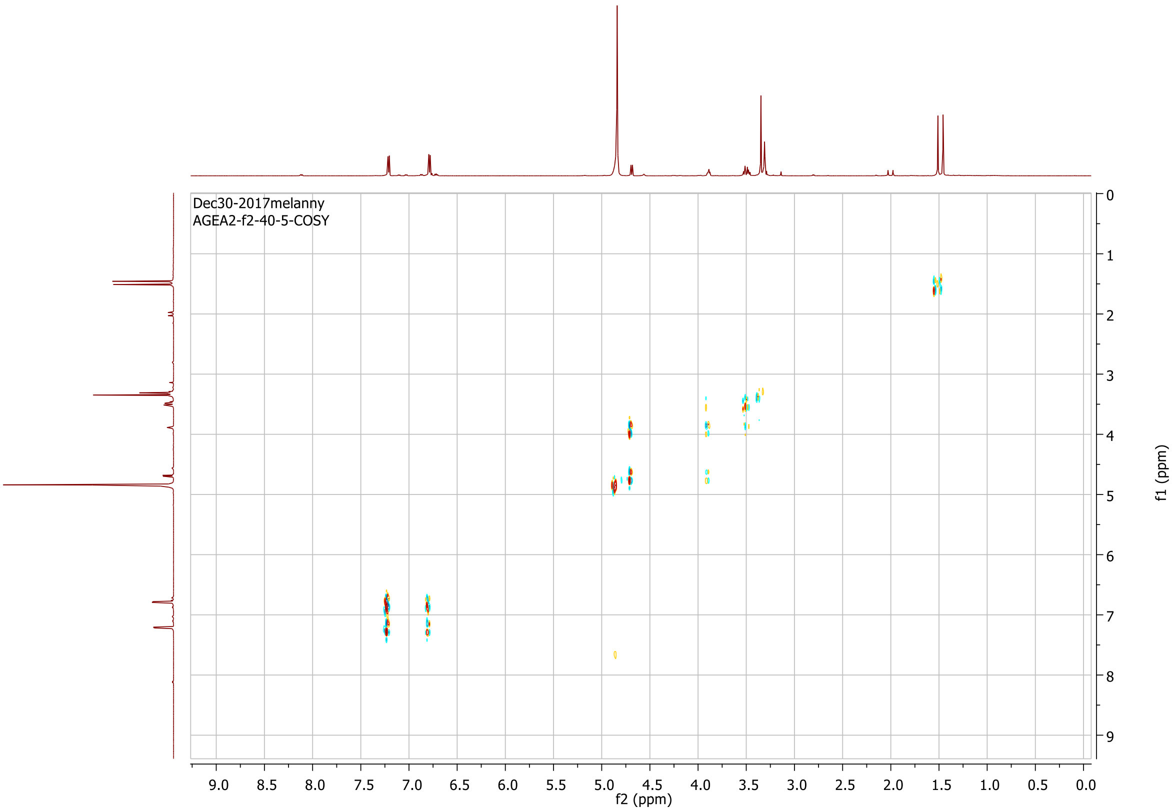

Figure S49 HSQC spectrum of 6

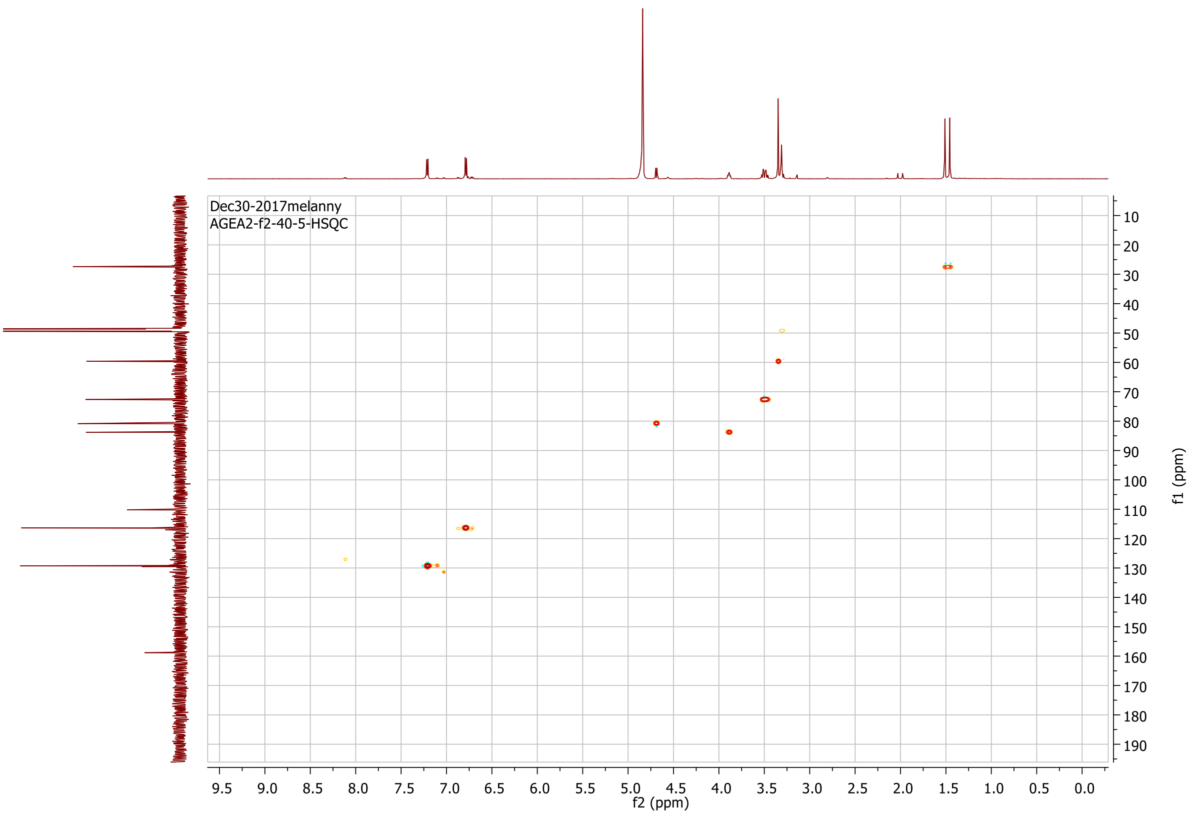

Figure S50 HMBC spectrum of 6

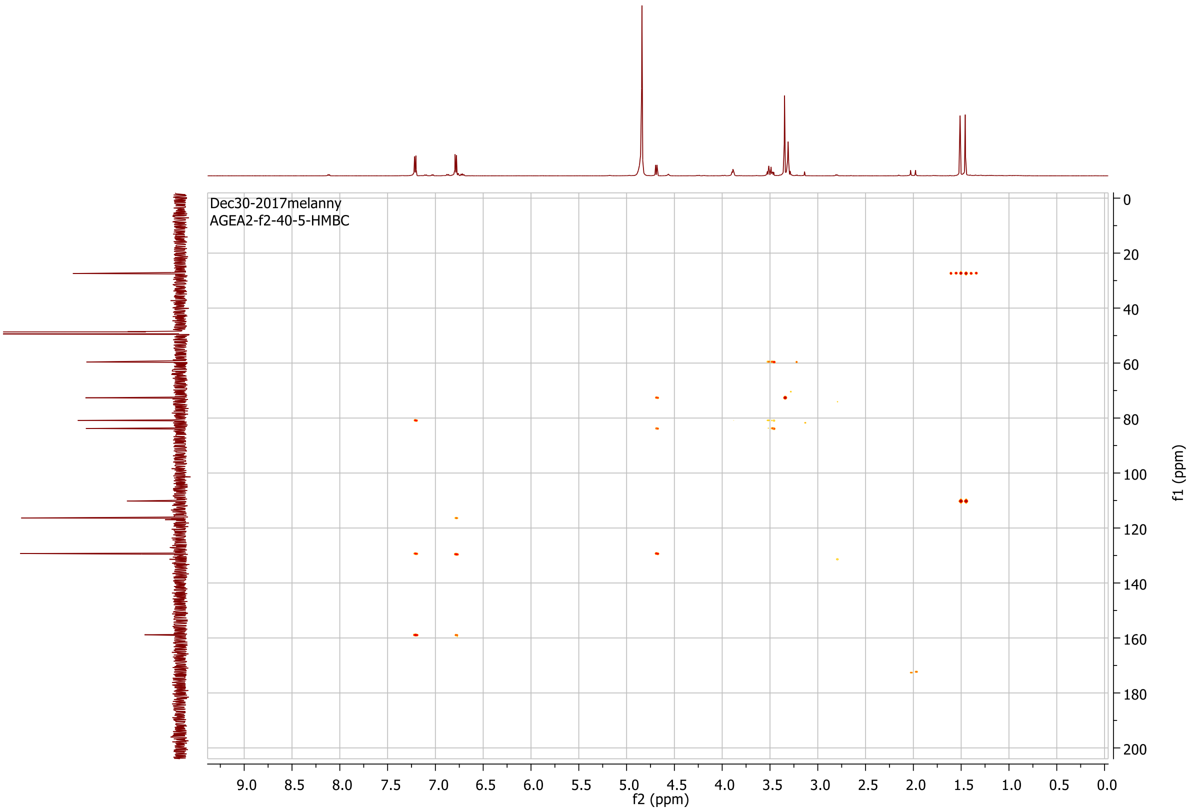

Figure S51 IR spectrum of 6

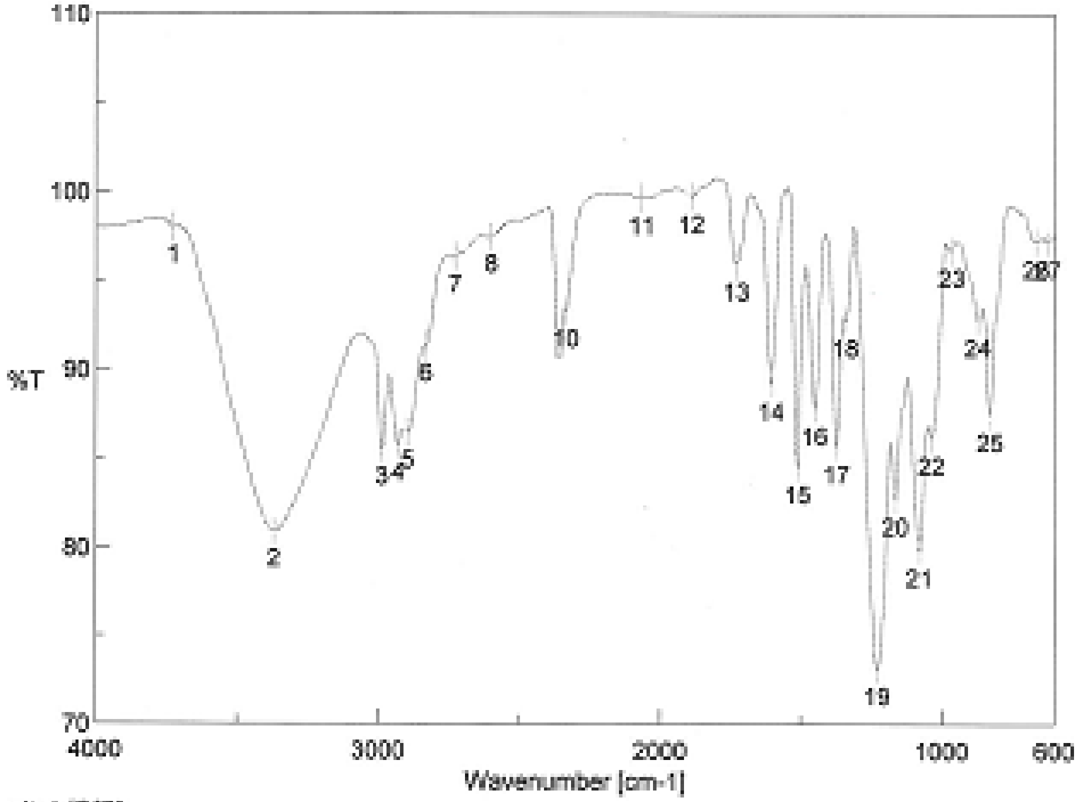

[コメント情報]
試料名
コメント
測定者
所属
会社 HU

[データ情報]		[測定情報]	
作成日時	2018/08/20 14:25	機種名	FT/IR-4600typeA
		シリアル番号	D015461786
データタイプ	等間隔データ		
横軸	Wavenumber [cm-1]	測定日時	2018/08/20 14:24
縦軸	%T		
スタート	599.753 cm-1	光源	標準光源
エンド	7800.65 cm-1	検出器	TGS
データ間隔	0.964233 cm-1	積算回数	10
データ数	7469	分解	4 cm-1
		ゼロフィリング	On
		アポダイゼーション	Cosine
		ゲイン	Auto (2)
		アパーチャー	Auto (7.1 mm)
		スキャンスピード	Auto (2 mm/sec)
		フィルタ	Auto (30000 Hz)

[ピーク検出結果]

No.	位置	強度	No.	位置	強度
1	3725.8	97.9921	2	3386.07	80.9102
3	2906.23	85.5728	4	2929.34	85.7724
5	2895.59	86.4058	6	2830.03	91.3661
7	2728.78	96.3806	8	2600.54	97.5193
9	2360.44	90.2334	10	2335.37	93.3024
11	2067.32	99.6625	12	1891.83	99.7419
13	1732.73	95.9512	14	1607.38	89.204

Figure S52 ^{1}H NMR spectrum of 7

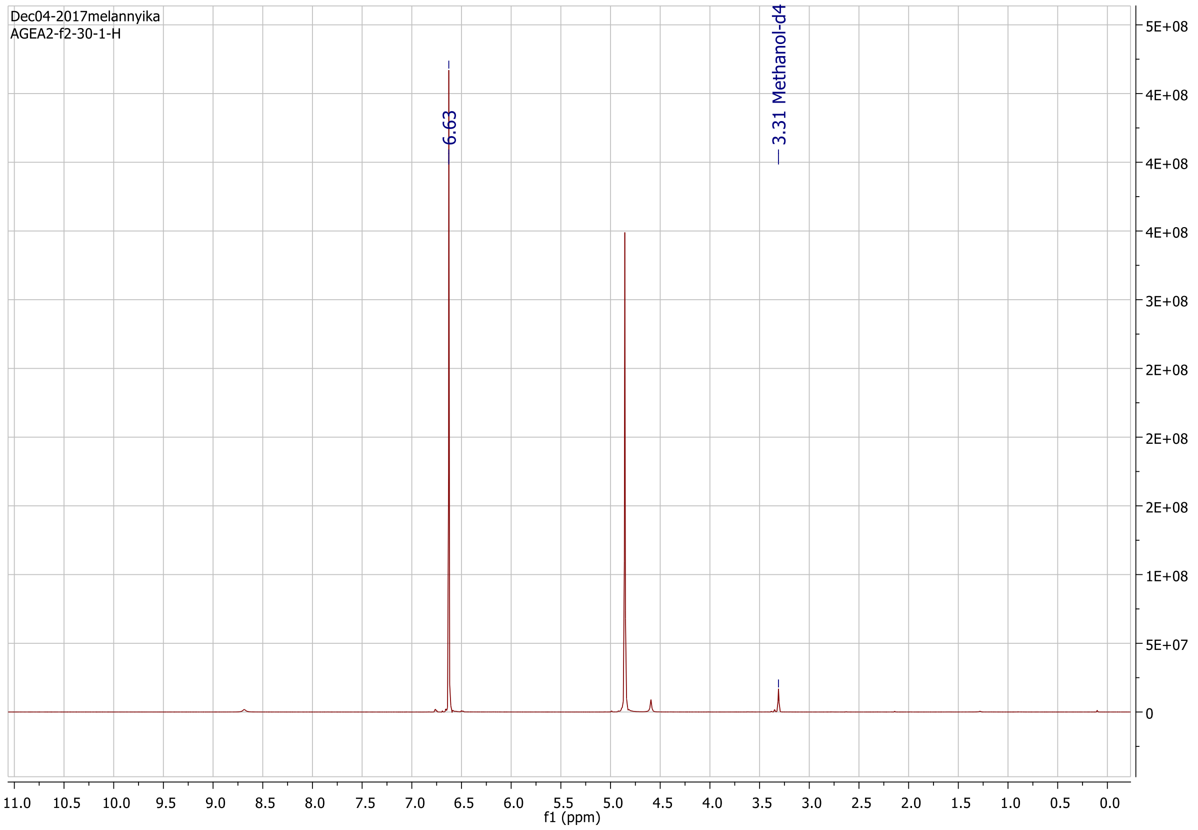

Figure S53 ^{13}C NMR spectrum of 7

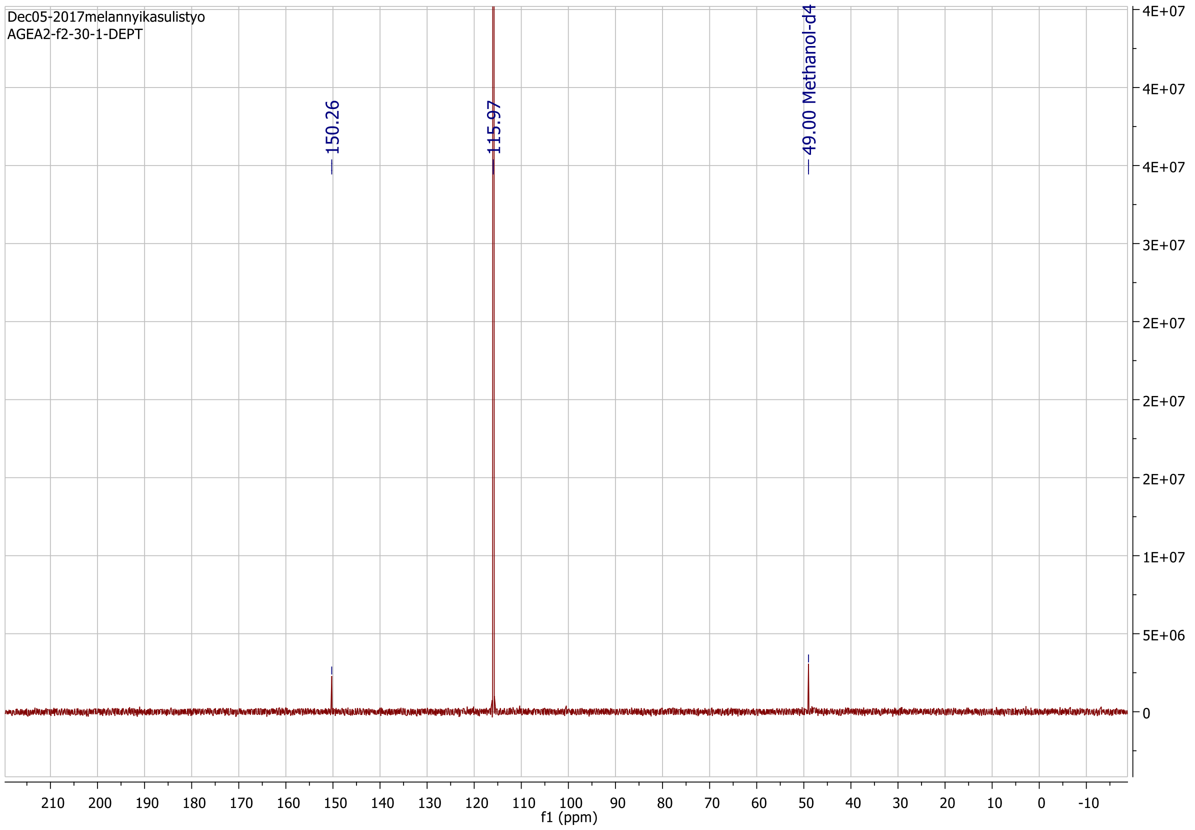

Figure S54 ¹H NMR spectrum of 8

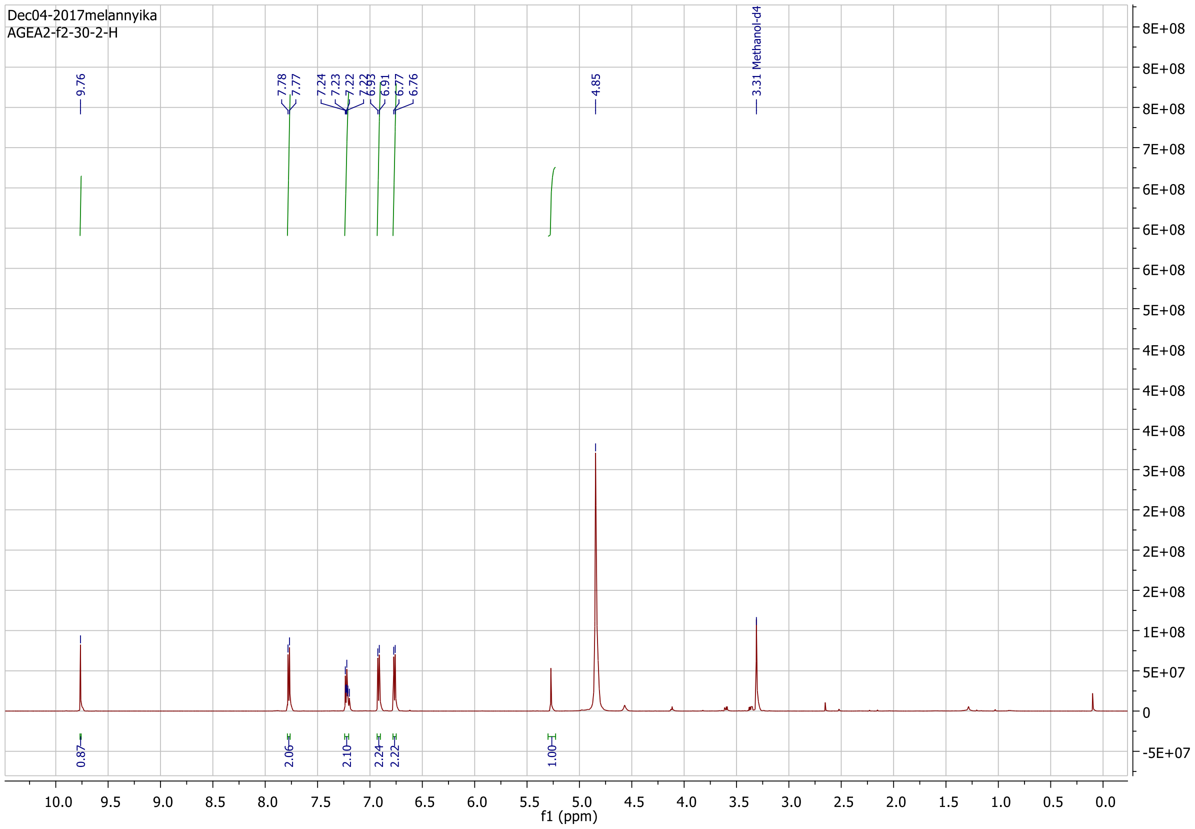

Figure S55 ^{13}C NMR spectrum of 8

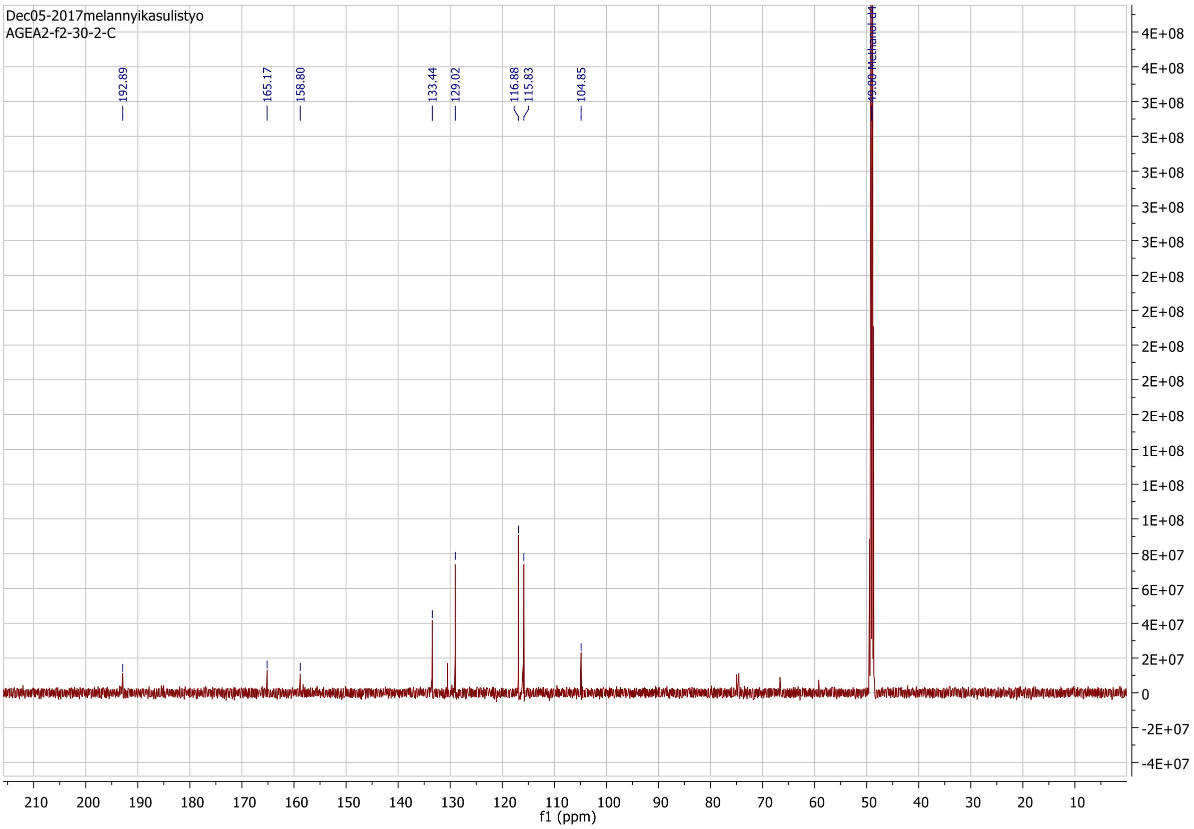

Figure S56 ^{1}H NMR spectrum of 9

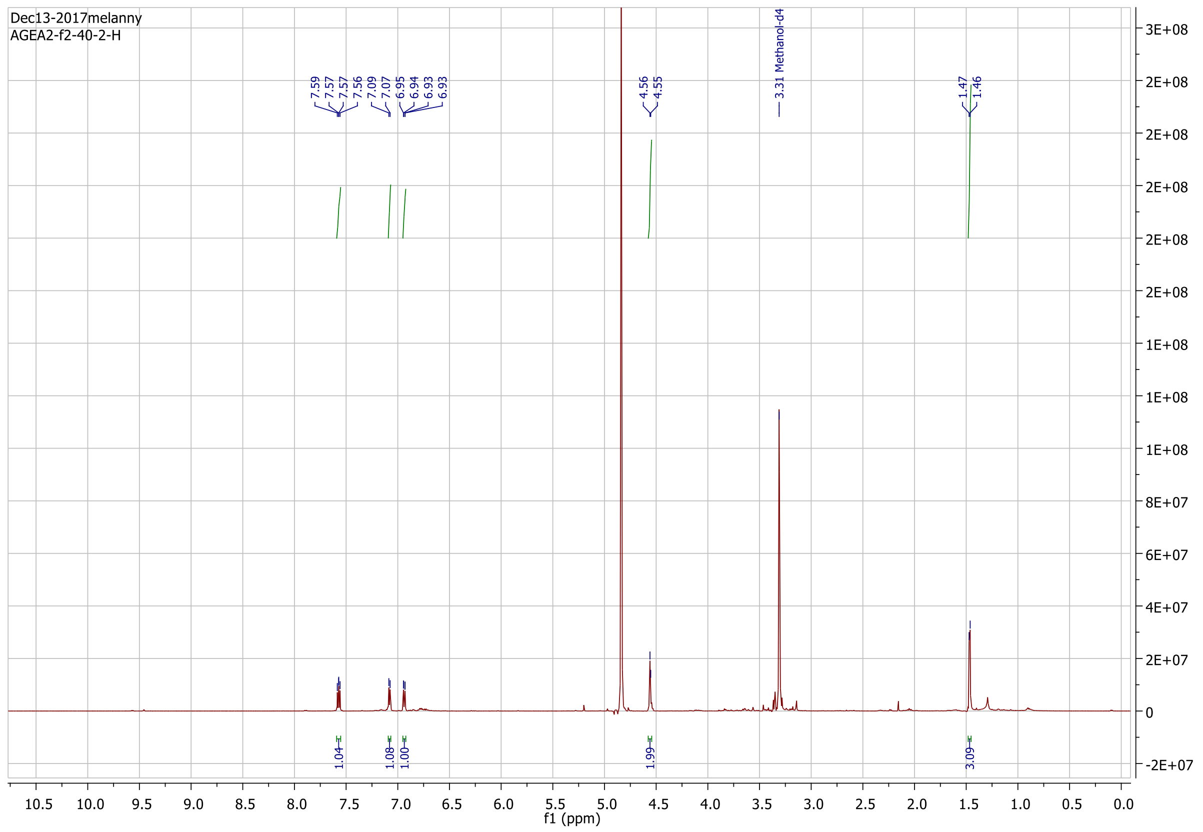

Figure S57 ^{13}C NMR spectrum of 9

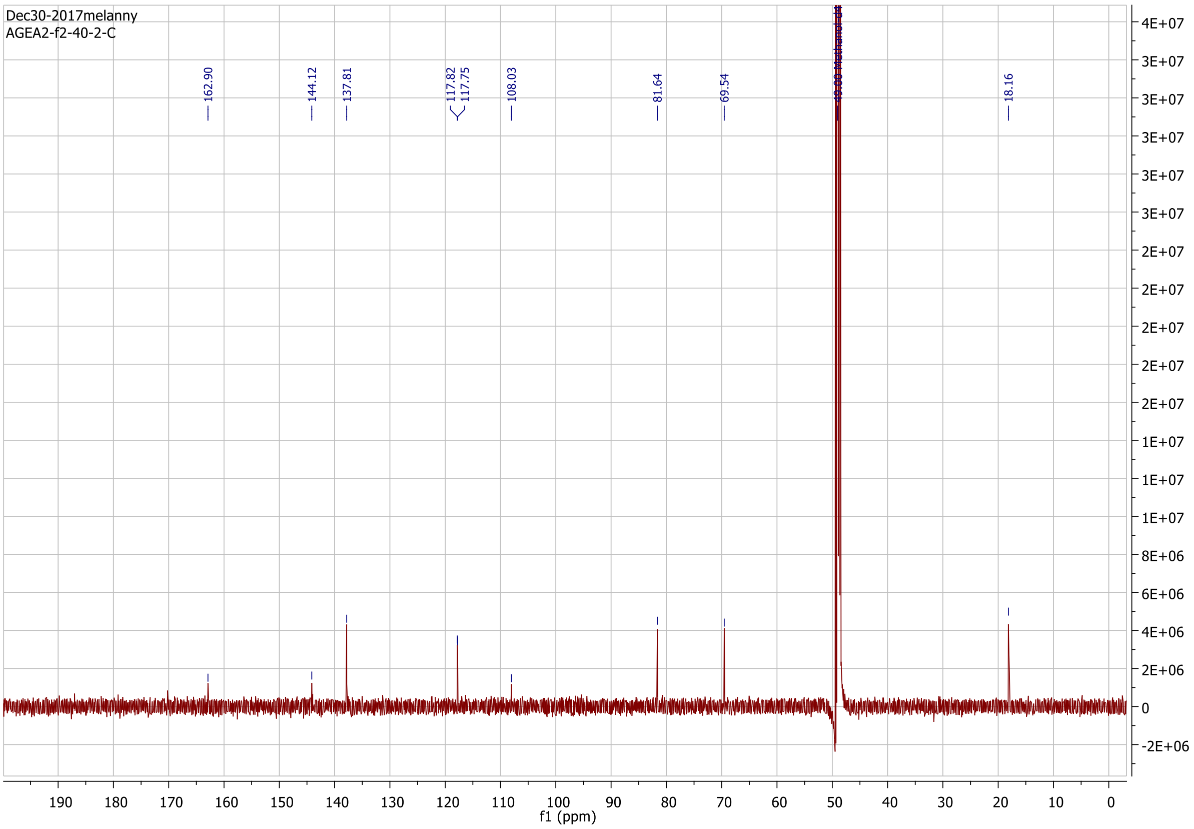

Figure S58 ^{1}H NMR spectrum of 10

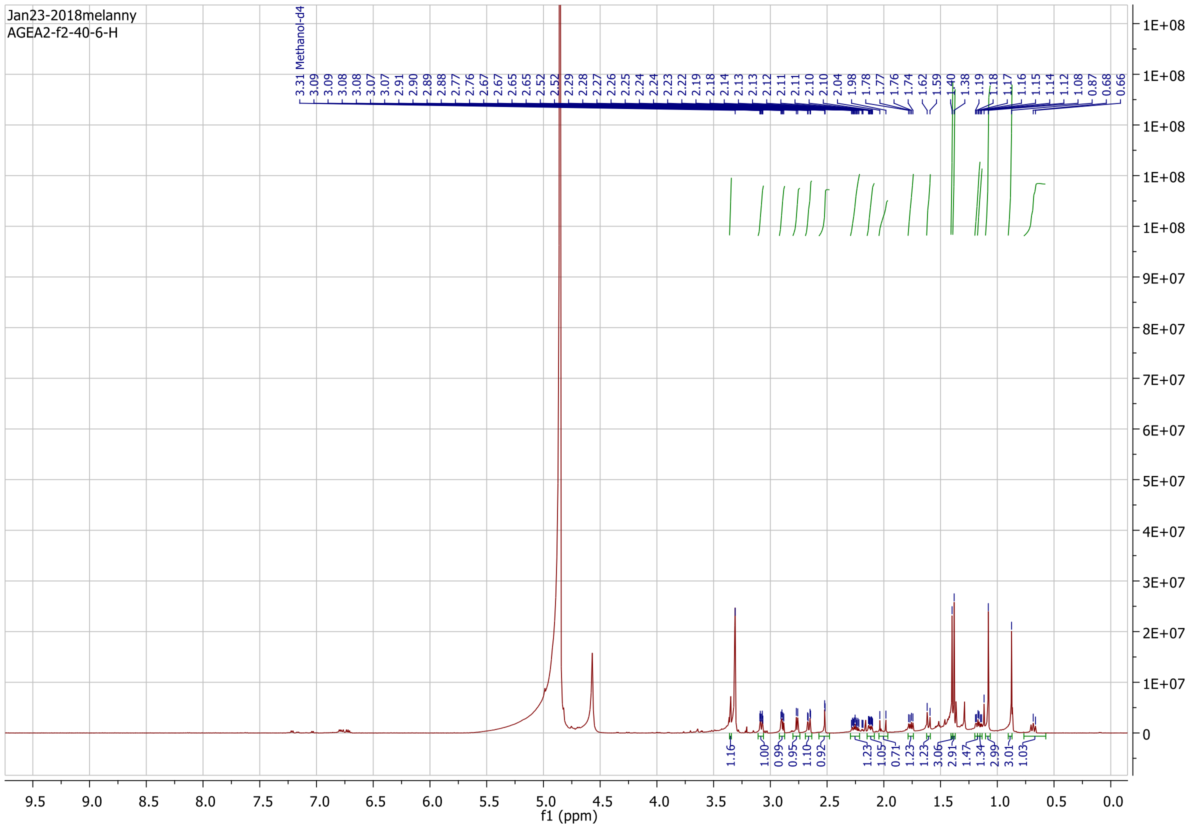

Figure S59 ^{13}C NMR spectrum of 10

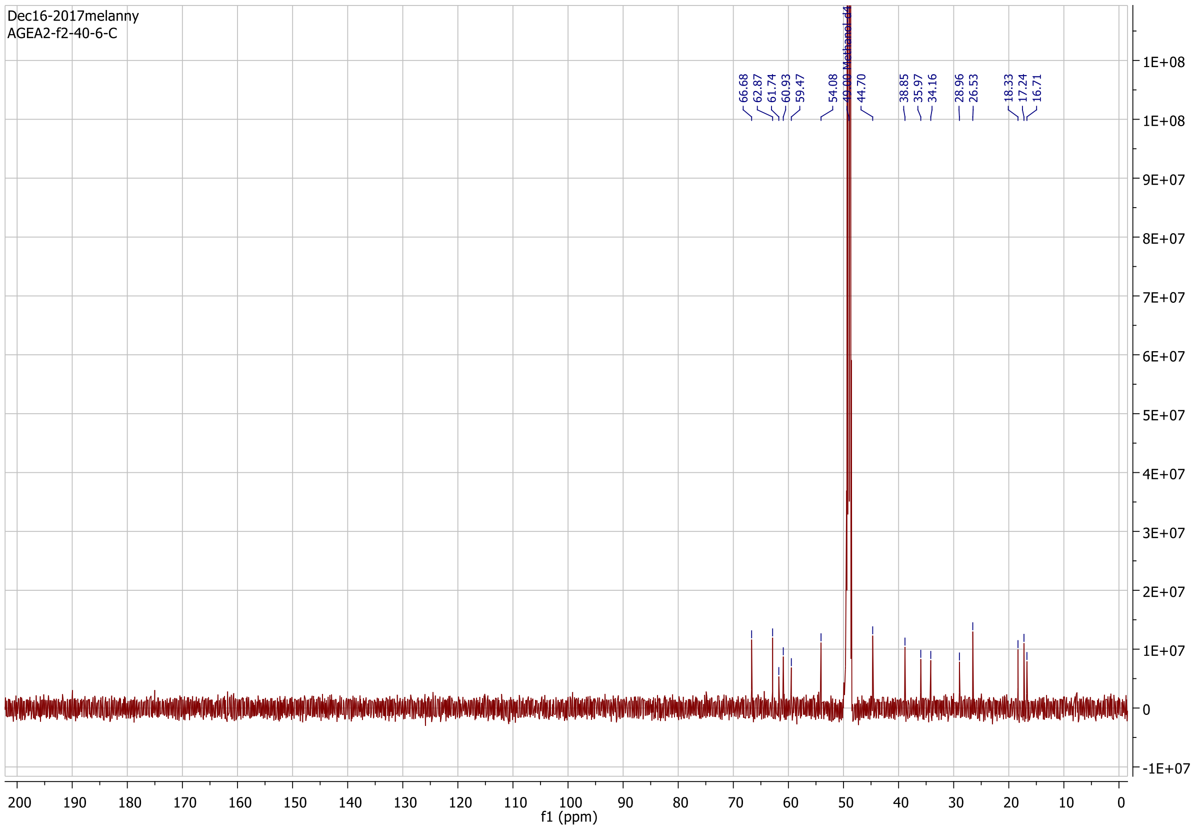

Figure S60 Structures of positive control for the bioassay

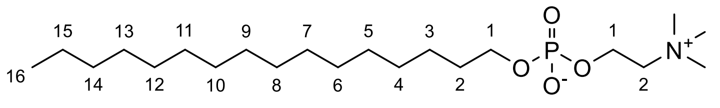

miltefosine

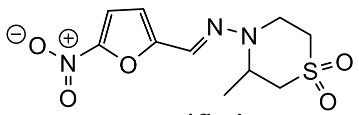

niflutimox

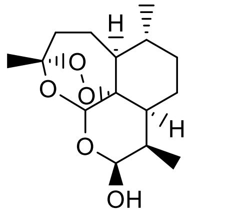

dihydroartemisinin

Table S1 COSY correlations of compounds 1-6

**Position**	Compound **1**	**2**	**3**	**4**	**5**	**6**
H-2, 6	H-3, 5	H-3, 5	H-3, 5	H-3, 5	H-3, 5	H-3, 5
H-3, 5	H-2, 6	H-2, 6	H-2, 6	H-2, 6	H-2, 6	H-2, 6
H-1′	H-2′	H-2′	H-2′	H-2′	H-2′	H-2′
H-1′-OCH_3	-	-	-	-	-	-
H-2′	H-1′, 3′	H-1′, 3′	H-1′, 3′	H-1′, 3′	H-1′, 3′	H-1′, 3′
H-3′	H-2′	H-2′	H-2′	H-2′	H-2′	H-2′

Table S2 HMBC correlations of compounds 1-6

**Position**	Compound **1**	**2**	**3**
H-2, 6	C-4, 6(2)	C-4, 6(2), 3(5), 1′	C-4, 6(2), 3(5), 1′
H-3, 5	C-1, 5(3), 4	C-1, 5(3), 4	C-1, 5(3), 4
H-1′	C-1, 2(6), 2′	C-1, 2(6), 2′, 3′, 1′-OCH_3	C-1, 2(6), 2′, 3′
H-1′-OCH_3	-	C-1′	-
H-2′	C-1, 1′	C-1, 1′, 3′	C-1, 1′, 3′
H-3′	C-1′, 2′, 3′-OCH_3	C-1′, 2′, 3′-OCH_3	C-1′, 2′, 3′-OCH_3
H-3′-OCH_3	C-3′	C-3′	C-3′

**Position**	Compound **4**	**5**	**6**
H-2, 6	C-4, 6(2), 3(5), 1′	C-4, 6(2), 3(5), 1′	C-4, 6(2), 3(5)
H-3, 5	C-1, 5(3), 4	C-1, 5(3), 4	C-1, 5(3), 4
H-1′	C-1, 2(6), 2′, 3′	C-1, 2(6), 2′, 1′-OCH_3	C-1, 2′, 3′
H-1′-OCH_3	-	C-1′	-
H-2′	C-1′, 3′	C-1	C-1, 1′
H-3′	C-1′,2′, 3′-OCH_3	C-1′, 2′, 3′-OCH_3	C-1′, 3′-OCH_3
H-3′-OCH_3	C-3′	C-3′	C-3′
H-2"	-	-	C-1′′
H-3"	-	-	C-1′′
